# Recent Progress on Flexible Multimodal Sensors: Decoupling Strategies, Fabrication and Applications

**DOI:** 10.1002/adma.202521375

**Published:** 2026-01-19

**Authors:** Tao Wu, Yu‐Tao Li, Luyu Zhao, Yi Zhang, Zhaojie Zhang, Jian Yuan, Yiwen Wu, Anqi Che, Yuanxiao Ma, Yang Chai, Yeliang Wang

**Affiliations:** ^1^ MIIT Key Laboratory For Low‐Dimensional Quantum Structure and Devices School of Integrated Circuits and Electronics Beijing Institute of Technology Beijing China; ^2^ The School of Information Science and Technology Beijing University of Chemical Technology Beijing China; ^3^ Department of Applied Physics The Hong Kong Polytechnic University Hong Kong China

**Keywords:** decoupling mechanism, flexible multimodal sensors, human‐machine interaction, signal decoupling

## Abstract

Flexible multimodal sensors have garnered significant attention in research areas such as electronic skin, advanced robotics, and personalized health monitoring due to their ability to leverage the complementary advantages of diverse sensing units, thereby a primary decoupling strategy exploits differences in the fundamental types of signals generated. Nevertheless, flexible multimodal sensors persistently face challenges like signal crosstalk and complex integration processes, which constrain their performance. This review delineates recent advances in flexible multimodal sensor decoupling through fundamental material design guided by physical principles, structural design, and AI‐driven signal decoupling architectures. Additionally, we explore the various applications of flexible multimodal sensors, encompassing environmental monitoring, physiological health tracking, human‐machine interaction, and robotic perception. Finally, the conclusion, challenges, and future perspectives for next‐generation flexible multimodal sensing systems are discussed.

## Graphical abstract

In this review, we establish a tripartite decoupling framework for flexible multimodal sensors, which elucidates the underlying principles of signal crosstalk and their solutions through material design, structural engineering, and AI algorithms. We also demonstrate its potential applications across environmental monitoring, health monitoring, human‐machine interaction, and intelligent robotics.

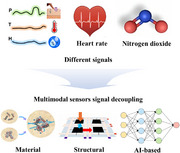

## 1 Introduction

The inherent rigidity, limited strain tolerance, and poor biocompatibility of conventional silicon‐based sensors restrict their utility in scenarios requiring large deformations or conformal contact with rough surfaces, driving the need for flexible sensors [1]. Recent years have witnessed growing interest in flexible sensors due to their applications in environmental monitoring, physiological health tracking, human‐machine interaction, and robotic perception [2, 3]. Unimodal flexible sensors are typically limited to detecting a single type of physical parameter, such as temperature [4], mechanics (pressure [5], shear force [6], and torsion [7]), humidity [8], and gas [9]. This functional limitation severely restricts their application in complex environments.

Consequently, with advancing research, scientists are shifting focus to multimodal sensors, which detect multiple parameters simultaneously [10]. These multimodal sensors typically leverage established sensing mechanisms, such as the thermoelectric [11, 12], piezoresistive [13, 14], piezocapacitive [15, 16], piezoelectric [17, 18], and triboelectric [19, 20] effects, often combining multiple of them within a single device. Flexible multimodal sensors detect and respond to changes in multiple physical properties simultaneously, meeting the demand for diverse physical parameter measurements in complex environments [21]. However, the research on multimodal sensors is currently hindered by signal coupling problems, such as temperature‐pressure coupling [22, 23], temperature‐humidity coupling [24, 25], multidirectional force coupling [26, 27], and other multiphysics coupling [28], which seriously restrict measurement accuracy and reliability [29]. Therefore, it is imperative to conduct comprehensive research on multimodal sensor decoupling.

Decoupling in multimodal sensors is usually enabled by a variety of materials and structures [30, 31]. The core approach fundamentally relies on distinct physical mechanisms to distinguish between mechanical stimuli through corresponding electrical signals (e.g., capacitance, resistance, current, or voltage). While materials and structural design provide significant advantages for multimodal sensor decoupling, inherent limitations such as nonlinear errors and environmental interference susceptibility persist. Specifically, material designs confront inherent trade‐offs between sensitivity and range, alongside vulnerability to environmental interference like temperature [32, 33, 34]. Structural strategies are limited by geometric complexity and challenging fabrication processes despite their isolation efficacy [35]. To overcome these challenges and achieve higher precision decoupling, the synergistic integration of artificial intelligence (AI) with multimodal sensors is emerging, ushering in the Sensing 4.0 era [36]. Driven by breakthroughs in computing, deep learning now dominates the field of artificial intelligence, utilizing multi‐layer neural networks to process massive datasets and provide high‐precision predictions and decisions [37]. Current AI‐driven approaches for decoupling multimodal signals have seen significant development, including machine learning (such as HSVM) [38], deep learning algorithms [39] (specifically LSTM) [40], and neural networks (such as CNN and RNN) [41]. Nevertheless, AI‐driven methods themselves face hurdles, including a heavy reliance on data, as well as challenges in model interpretability, generalizability, and edge deployment [42, 43]. Overcoming these hurdles is key to unlocking transformative advances for next‐generation intelligent flexible sensing systems.

**FIGURE 1:**
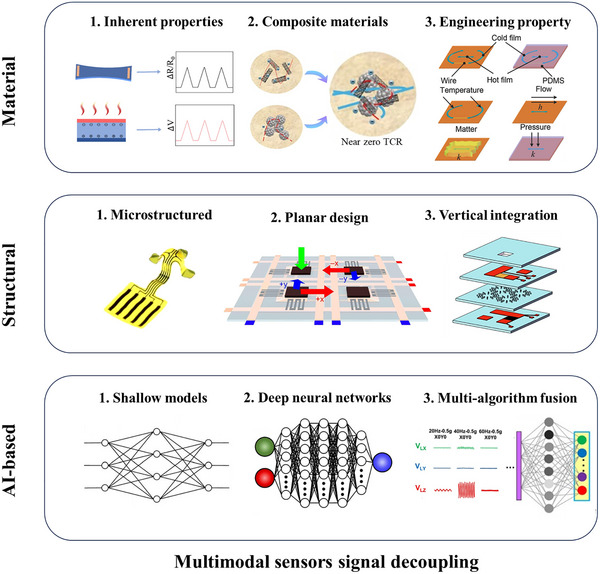
Overview of multimodal sensor decoupling principle. Material design decoupling. Reproduced with permission [57]. Copyright 2024, American Chemical Society. Reproduced with permission [44]. Copyright 2024, Elsevier Ltd. Reproduced with permission [45]. Copyright 2020, The American Association for the Advancement of Science. Structure design decoupling. Reproduced with permission [46]. Copyright 2019, American Chemical Society. Reproduced with permission [47]. Copyright 2024, Elsevier lnc. Reproduced with permission [35]. Copyright 2024, American Chemical Society. AI‐based signal decoupling. Reproduced under terms of the CC‐BY Creative Commons Attribution 4.0 International License (http://creativecommons.org/licenses/by/4.0/) [48]. Copyright 2013, Feilu Wang, published by IFSA. Reproduced with permission [49]. Copyright 2023, WILEY‐VCH GmbH. Reproduced with permission [50]. Copyright 2023, American Chemical Society.

**FIGURE 2:**
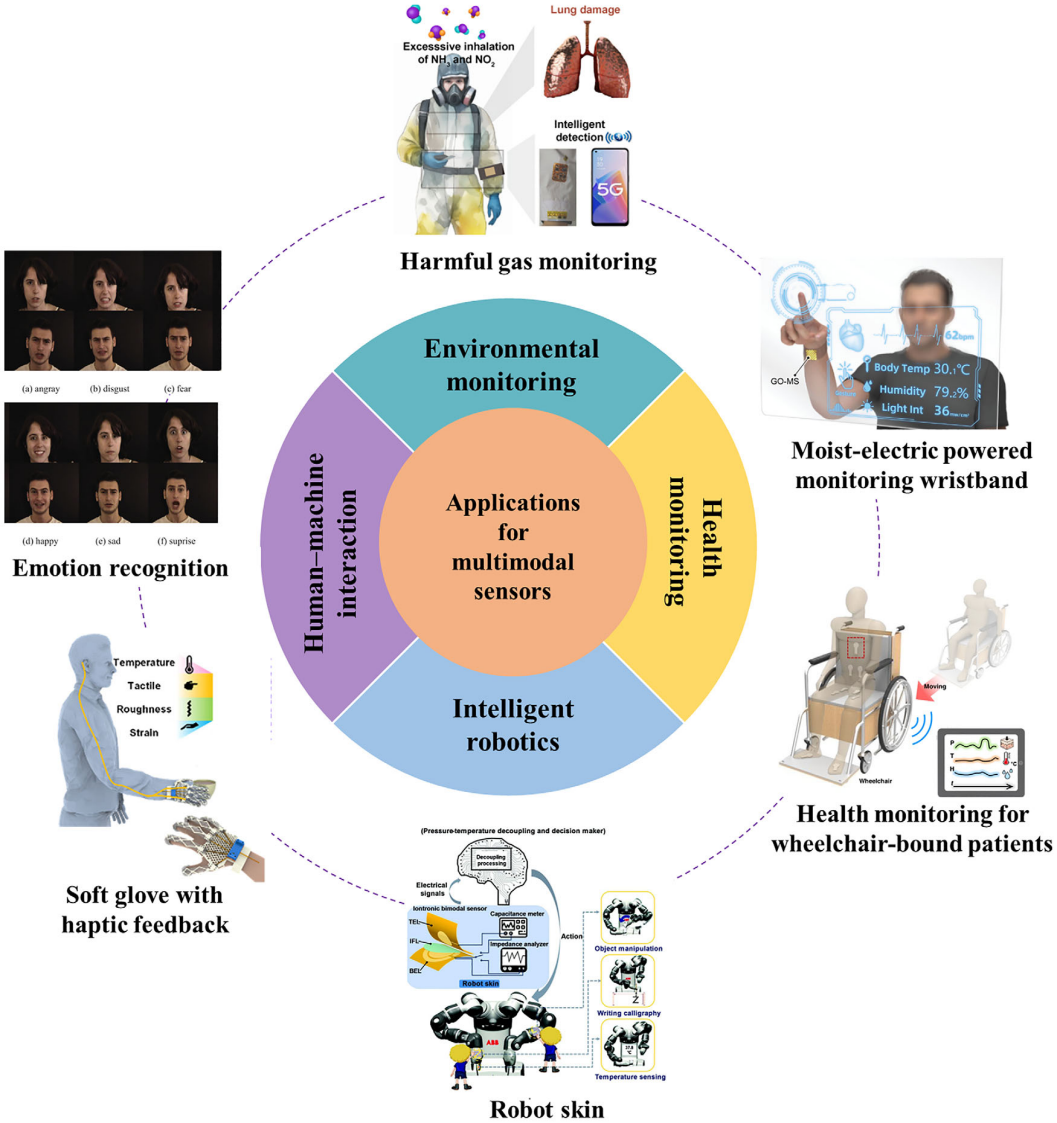
Overview of multimodal sensor applications. Harmful gas monitoring. Reproduced with permission [51]. Copyright 2024, John Wiley and Sons. Moist‐electric powered monitoring wristband. Reproduced with permission [52]. Copyright 2022, Wiley‐VCH GmbH. Health Monitoring for patients seated in wheelchairs. Reproduced under terms of the CC‐BY Creative Commons Attribution 4.0 International License (http://creativecommons.org/licenses/by/4.0/) [53]. Copyright 2023, Seokjoo Cho, published by Springer Nature. Robot skin. Reproduced with permission [54]. Copyright 2023, WILEY‐VCH GmbH. Soft glove with haptic feedback. Reproduced with permission [55]. Copyright 2022, American Chemical Society. Emotion recognition. Reproduced with permission [56]. Copyright 2020, Elsevier B.V.

In this review, we systematically present the three primary approaches for decoupling multimodal sensors, summarize their application scenarios and integration fabrication techniques, and discuss the associated challenges and future perspectives. As illustrated in Figure 1, three primary approaches enable the decoupling of multimodal sensors. First, material design, which fundamentally achieves signal separation by strategically leveraging distinct material properties through synergistic intrinsic mechanisms and property engineering, offering the advantage of inherent decoupling at the foundational level. Second, structural design refines the sensor's physical architecture using hardware‐level mechanisms (e.g., geometric separation, zonal layout, localized reinforcement) to enhance targeted responses while suppressing interference, providing the advantage of robust physical isolation and tailored sensitivity. Third, AI‐driven decoupling employs advanced computational algorithms (such as HSVM and DNN) to actively reconstruct intrinsically coupled signals into separable representations, offering the significant advantage of achieving high‐precision decoupling even for complex signal interactions, thereby substantially improving multi‐sensory data interpretation accuracy. Then, as presented in Figure 2, the applications of flexible multimodal sensors in fields of environment monitoring, physiological health monitoring, human‐machine interaction (HMI), and intelligent robotics are exemplified. Looking ahead, flexible multimodal sensors hold immense potential to revolutionize domains including human‐machine collaboration and autonomous robotics, ultimately serving as a cornerstone for next‐generation intelligent systems that drive technological transformation and societal progress.

## 2 Material Design Decoupling

Despite significant progress in multimodal sensor development, the effective signal decoupling remains a major challenge. Early research primarily achieved signal separation through multifunctional materials, but its effectiveness was fundamentally limited by inherent multiphysics interference within material systems. To actively suppress these coupling effects, current strategies focus on synergistic material design exploiting intrinsic physical mechanisms, such as leveraging inherent decoupling properties (e.g., Seebeck effect vs. piezoresistance), composite materials design (e.g., opposing thermal expansion coefficient or resistance temperature coefficient), and property‐engineered decoupling (e.g., thermal conductivity gradients or magnetic anisotropy gradient).

### 2.1 Inherent Material Properties

Certain materials inherently decouple signals through their distinctive physical properties and signal generation mechanisms. This strategy embeds decoupling directly into the material's response, circumventing the need for complex post‐processing algorithms. It fundamentally capitalizes on the orthogonality inherent to the material system, which can manifest in different signal types (e.g., resistance vs. voltage), distinct generation principles (e.g., piezoresistive vs. thermoelectric), or differentiable response signatures, achieving separation at the source.

**FIGURE 3:**
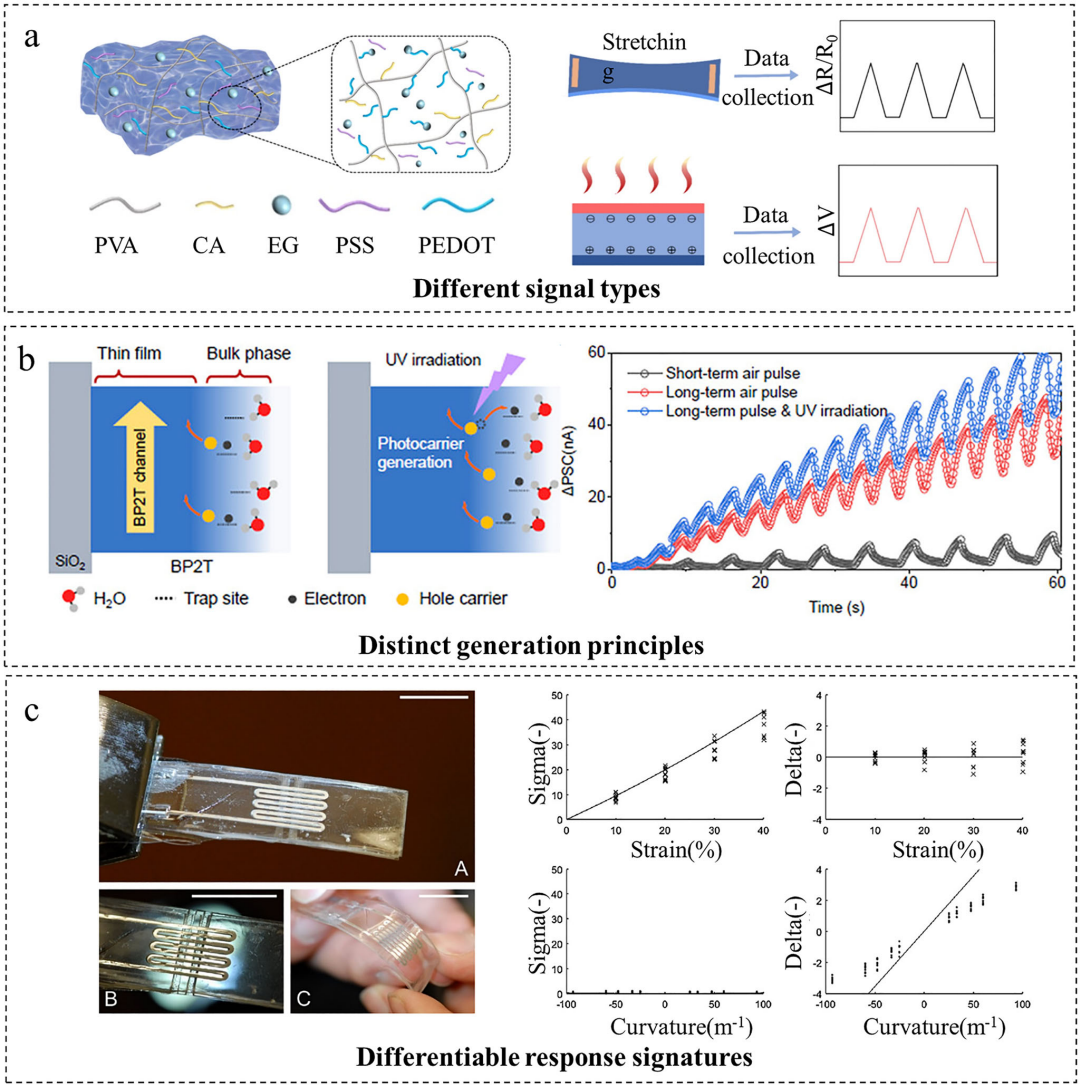
Decoupling through inherent decoupling properties. (a) Dual sensing mechanisms of PEDOT:PSS: resistance response to strain and voltage response to temperature gradients. Reproduced with permission [57]. Copyright 2024, American Chemical Society. (b) Dual generation mechanisms via phase separation in a BP2T synaptic sensor. Reproduced under terms of the CC‐BY Creative Commons Attribution 4.0 International License (http://creativecommons.org/licenses/by/4.0/) [59]. Copyright 2025, John Wiley and Sons. (c) Symmetrically configured liquid metal units for decoupling: summation (Σ) yields strain, while difference (Δ) yields curvature. Reproduced with permission [63]. Copyright 2016, Elsevier B.V.

A primary decoupling strategy exploits differences in the fundamental types of generated signals. Zhu et al. [57] achieved inherent isolation of strain and temperature signals through synergistic material design and physical mechanisms. As shown in Figure 3a, PEDOT:PSS leverages its dual sensing mechanisms, where deformation of the conductive network produces a resistance response to strain, and the Seebeck effect yields a voltage response to temperature gradients. These two signals are naturally decoupled in terms of physical nature (resistance vs. voltage). Ultimately, the sensor achieves bidirectional independent monitoring within the ranges of 20°C–100°C and ≤200 kPa. This strategy is equally potent for environmental sensing. Kan et al. [58] developed a sensing unit that simultaneously outputs resistive and capacitive signals, leveraging this inherent orthogonality to ensure the NO_2_ response dominates the resistive channel while humidity modulates the capacitive channel. This enabled interference‐free NO_2_ detection across 11%–95% relative humidity without algorithmic compensation.

Decoupling can also originate from fundamentally different generation principles within a material. Mao et al. [59] developed a bimodal synaptic sensor from a dual‑phase organic semiconductor (BP2T). As shown in Figure 3b, its distinct bulk and channel phases independently sense respiratory humidity via charge trapping and UV light via photosensitivity. This principle extends to multi‑parameter arrays through selective material chemistry. Li et al. [60] constructed such an array by coaxial wet‑spinning, integrating specific ionophores for Na^+^, K^+^, Ca^2^^+^, and a H^+^‑sensitive polyaniline into composite yarns. Each sensing unit responds exclusively to a target sweat biomarker or temperature, enabling synchronous and independent multi‑signal monitoring in a wearable system, which demonstrated high sensitivity (e.g., 39.52 ± 0.5 mV/pH for pH) and long‑term stability (signal drift <0.2 mV/h).

The third pathway leverages differentiable signatures in the material's response, which often manifest in the temporal or spatial domain to distinguish stimuli that engage similar generation mechanisms. Guo et al. [61] developed a self‐powered capacitive interface where both pressure and ambient humidity modulate moisture‐induced electrical double layers. Decoupling is achieved by their intrinsically distinct temporal signatures: sharp transient peaks for pressure vs. slow drifts for ambient variations. Alternatively, the spatial layout of sensing elements can be engineered to generate distinct response patterns. For instance, Roberts et al. [62] realized this by designing an elastomer sheet embedded with fluidic parallel‐plate capacitors, where pressing and shearing induce electrode convergence or lateral sliding, respectively. The resulting differential capacitance changes across the capacitor array allow shear (up to ±3 mm) and pressure (up to 25 kPa) to be independently resolved. Similarly, as shown in Figure 3c, White et al. [63] fabricated micro channels in Sylgard 184 via laser ablation, utilizing gallium‐indium alloy as sensing elements. They achieved intrinsic decoupling of strain and curvature through symmetrically paired back‐to‐back liquid metal strain units. Under uniform substrate stretching, both units exhibited synchronous resistance variations, with their sum (Σ) characterizing strain. Under substrate bending, the units showed opposite resistance changes, with their difference (Δ) indicating curvature direction and magnitude. By isolating Σ/Δ signals in real‐time and applying theoretical models, strain and curvature were directly output with cross‐coupling errors an order of magnitude below primary signals.

This strategy achieves signal separation at the source by capitalizing on the intrinsic properties of materials, which enable orthogonal signal types, distinct generation principles, or differentiable response signatures. Its primary advantage lies in the conceptual elegance and simplified readout enabled by this built‐in decoupling. However, its applicability is contingent upon the availability or design of materials possessing such orthogonal intrinsic responses to the target stimuli, which can limit generality.

### 2.2 Composite Materials Design

When inherent material properties are insufficient for signal orthogonality, composite material design emerges as a core strategy for decoupling. This approach relies on a sophisticated multi‑component design to isolate signals at the physical source, typically by either combining components with opposing responses to cancel interference (e.g., temperature) or by creating physically or electrically isolated pathways that allow different signals to be transmitted and detected independently.

**FIGURE 4:**
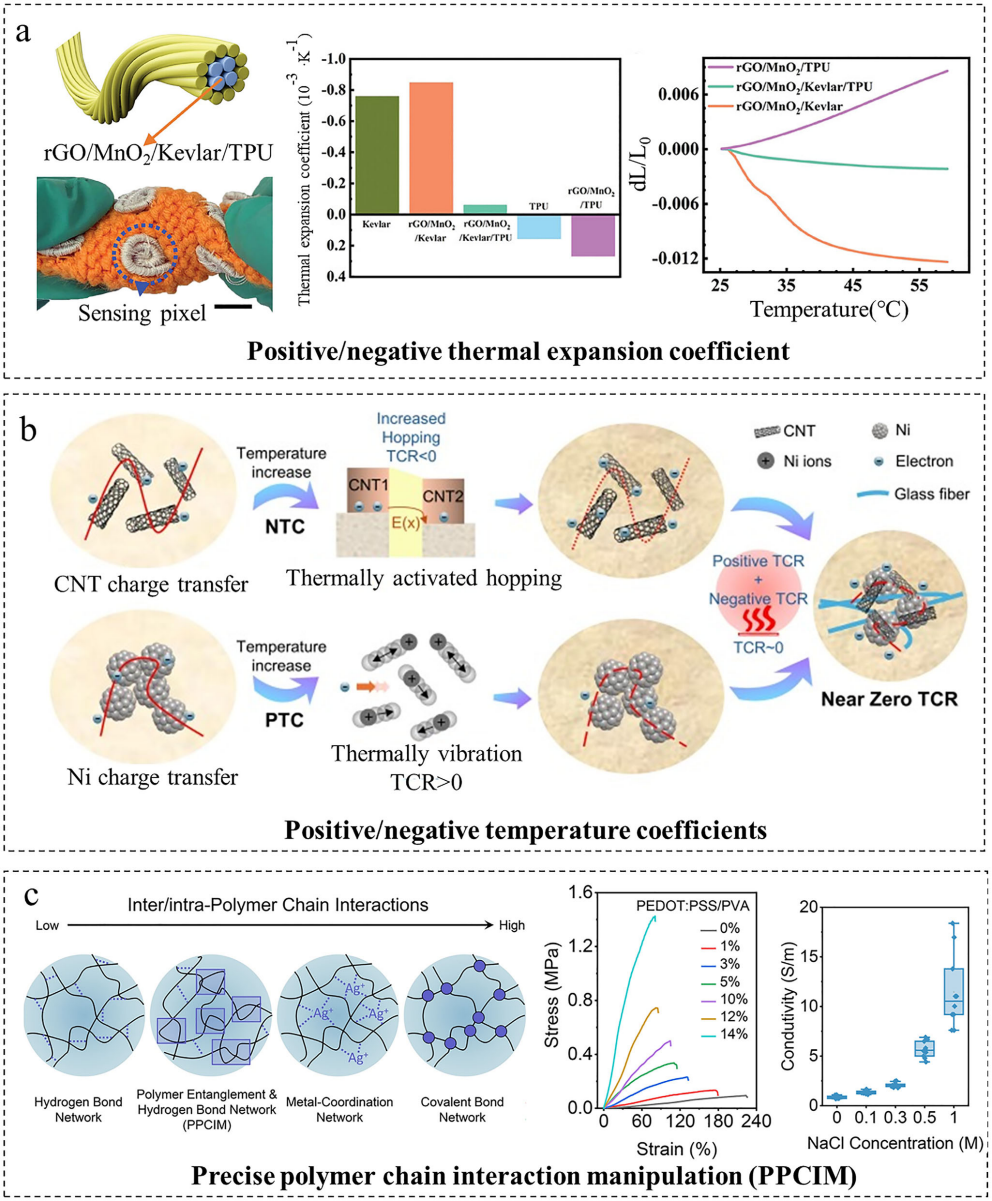
Decoupling through composite materials design. (a) Positive/negative thermal expansion coefficient materials design for decoupled temperature/pressure sensing. Reproduced with permission [64]. Copyright 2024, Wiley‐VCH GmbH. (b) Positive/negative temperature coefficient materials design for decoupled temperature/pressure sensing. Reproduced with permission [44]. Copyright 2024, Elsevier Ltd. (c) Precise inter‐/intra‐polymer‐chain‐interaction manipulation (PPCIM) for decoupled mechanical/electrical properties in composite hydrogels. Reproduced with permission [65]. Copyright 2025, Elsevier Ltd.

A direct application of the first pathway is to composite materials with opposite physical responses to nullify specific interfering stimuli. For example, by precision proportioning composites with positive/negative thermal expansion coefficient (TEC), this approach eliminates temperature‐induced interference with primary sensing signals and enables fundamental temperature/pressure decoupling. As illustrated in Figure 4a, Zhang et al. [64] fabricated a polymer matrix (rGO/MnO_2_/Kevlar/TPU) with a near‐zero thermal expansion coefficient by blending TPU (positive TEC) and Kevlar (negative TEC). This design eliminates irreversible damage to the conductive network caused by deformation under temperature fluctuations, thereby achieving strain‐insensitive temperature sensing. Meanwhile, pressure detection relies on the triboelectric nanogenerator (TENG) principle. The significant impedance difference between the high‐impedance TENG (≈300 MΩ) and the temperature‐sensing resistance (<10 MΩ) ensures that resistance variations induced by temperature minimally affect TENG’ s pressure‐electrical output. This material design strategy and integrated approach enable crosstalk‐free dual‐modal simultaneous monitoring of temperature and pressure.

Similarly, Zhang et al. [44] developed a flexible integrated sensor for dual temperature/pressure sensing. As depicted in Figure 4b, the pressure‐sensing element utilizes a temperature‐insensitive CNT/Ni/PVP/GF piezoresistive film that achieves its insensitivity through the opposing resistance temperature coefficients of nickel (Ni) and carbon nanotubes (CNT), thereby eliminating temperature‐induced interference in pressure signals. For temperature sensing, the CNT/GP/TPU composite (with substantially higher elastic modulus than the glass fiber substrate) leverages its significant strain coefficient mismatch with the substrate to precisely position temperature‐sensing points at the center of circular IDEs. This strategic placement effectively minimized pressure coupling interference. Consequently, the integrated sensor achieved bidirectional decoupling within 20°C–100°C and ≤200 kPa operational ranges.

A more sophisticated strategy entails constructing and governing independent functional networks within a composite system for orthogonal performance optimization. As shown in Figure 4c, Guo et al. [65] implemented this via precise polymer chain interaction manipulation (PPCIM) in a PVA/PEDOT:PSS/plasticizer hydrogel. By tailoring intermolecular interactions, they created separate networks governing mechanical integrity and electrical conduction, enabling decoupled optimization of these properties. In addition, decoupling is achieved by integrating multiple sensing functionalities into a unified yet physically isolated material platform. Xiang et al. [66] developed a hard‑magnetic graphene nanocomposite (HMGN) by infiltrating a Nd‑Fe‑B/PDMS mixture into a laser‑induced porous graphene network. On this single platform, ion‑selective membranes enable potentiometric detection, the doped graphene structure supports electrophysiological and impedance sensing, and the temperature‑dependent network allows thermal monitoring, ensuring all modalities operate without cross‑talk at the material level.

This approach designs composite materials to nullify specific interferences passively or to create physically/electrically isolated pathways for signals, often by incorporating components with counteracting properties (e.g., opposite thermal responses) or designing structural/electrical anisotropy. Its key strength lies in high design flexibility and effectiveness against strong common interferences like thermal drift, though it faces the challenge of optimizing complex multi‑component interactions.

### 2.3 Engineering of Property Differentials

Beyond leveraging intrinsic properties or composite materials, decoupling multimodal signals can be further advanced through engineering property differentials. This approach creates designed spatial or temporal gradients (e.g., in thermal conductivity, magnetic anisotropy) within a functionally graded medium. Different stimuli selectively modulate distinct aspects of the gradient, enabling a single transducer to output separable, multidimensional response patterns for physical‐level discrimination or computational demultiplexing.

**FIGURE 5:**
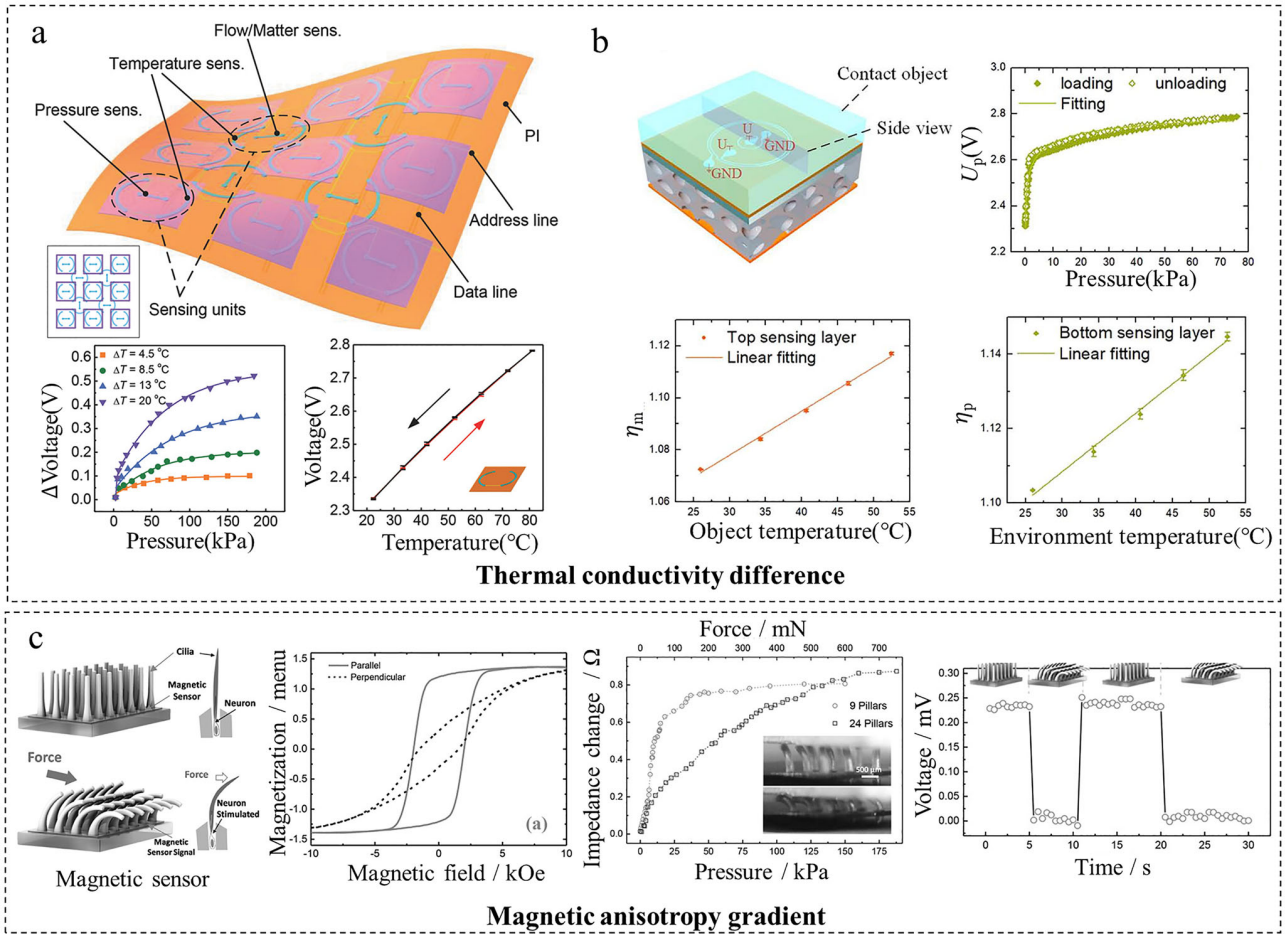
Decoupling through property‐engineered decoupling. (a) Decoupling temperature and stress based on thermal conductivity differences. Reproduced with permission [67]. Copyright 2017, WILEY‐VCH GmbH. (b) Thermal conductivity difference decoupling quadruple sensing. Reproduced with permission [45]. Copyright 2020, The American Association for the Advancement of Science. (c) Magnetic anisotropy gradient decoupling. Reproduced with permission [68]. Copyright 2015, WILEY‐VCH GmbH.

A quintessential implementation is the construction of a thermal conductivity gradient. As shown in Figure 5a, pioneering work by Zhao et al. [67] established a thermosensation‐based e‐skin using uniform platinum ribbons, where a single sensing element transduced pressure (via the piezothermic effect of a porous PDMS layer), material type (via thermal conductivity), and wind flow (via convection) into thermal signals. This design achieved high sensitivity across modalities, with a pressure detection limit of 0.5 kPa and a fast temperature response time of 8 ms, demonstrating robust performance under repeated mechanical deformation. Building on this foundation, their subsequent work developed a quadruple tactile sensor integrating pressure, material thermal conductivity, object temperature, and ambient temperature detection [45]. As shown in Figure 5b, the sensor uses two PDMS/AgNP layers featuring concentric Cr/Pt rings (hot/cold films). Hot films (central low‐resistance rings) enable material identification via thermal conduction differences and pressure sensing through porous PDMS deformation. Cold films (outer rings) monitor dual temperatures while compensating for thermal drift. This design enables real‐time object grasping across diverse orientations while achieving 94% classification accuracy in identifying seven categories of garbage for waste sorting tasks.

Similarly, the introduction of magnetic anisotropy gradients allows decoupling of force and flow by exploiting the directional dependence of magnetic response in aligned nanocomposites. For example, Alfadhel et al. [68] developed a magnetic nanocomposite cilia tactile sensor. As shown in Figure 5c, the sensor consists of highly elastic polydimethylsiloxane (PDMS) cilial structures containing permanently magnetic iron nanowires (NWs) aligned along their vertical axis, integrated atop a giant magneto‐impedance (GMI) magnetic sensor. The permanent magnetic moment of the NWs provides a stable bias field. When the cilia are deflected by an external force or fluid flow, the resulting change in the orientation and distribution of the magnetic field is detected by the GMI sensor as an impedance change. This design enables the sensor to achieve a high sensitivity of up to 214 mΩ mN^−1^, an ultrahigh resolution of 16 Pa, and an extremely low power consumption of 80 nW. It demonstrates multifunctional capabilities for detecting texture, vertical pressure, fluid flow, and even physiological signals like heart rate, operating in both air and water environments.

This strategy works by creating spatial or temporal gradients (e.g., in thermal or magnetic properties), where different stimuli selectively act on distinct gradient aspects. A single transducer can thus output multidimensional response patterns for computational demultiplexing. Although highly effective for achieving multifunctionality with minimal hardware and for decoupling combined stimuli, it generally relies on sophisticated device design and advanced signal processing.

Despite the successful utilization of intrinsic physical phenomena, composite design, and engineered material properties for signal isolation, these strategies remain limited by inherent constraints in intricate multi‐component optimization and reliance on complex device architectures coupled with advanced signal processing, respectively. To overcome these challenges, structural design offers a promising alternative to achieve interference‐free multimodal sensing.

## 3 Structural Design Decoupling

For decoupling in multimodal sensors, structural design‐based strategies provide a viable alternative to the aforementioned material‐based approaches, delivering a more direct route to interference reduction [21]. The essence of structural decoupling methods lies in isolating coupled interference from different physical signals through geometrically differentiated designs such as cages [46], frustums [69], pyramids [70, 71], pillars [72, 73], domes [74, 75, 76], wrinkle structure [77], and 3D architectures [78]. These structures facilitate signal separation via physically isolating sensing pathways (e.g., using cage legs or frustum arrays decoupling pressure and shear forces), positioning sensitive elements in deformation‐minimized zones (e.g., height‐differential layouts that position strain sensors away from pressure‐induced interference), and suppression of parasitic coupling via localized stiffening (e.g., using embedded rigid supports to prevent strain transmission to pressure sensors).

### 3.1 Microstructured Architectures

**FIGURE 6:**
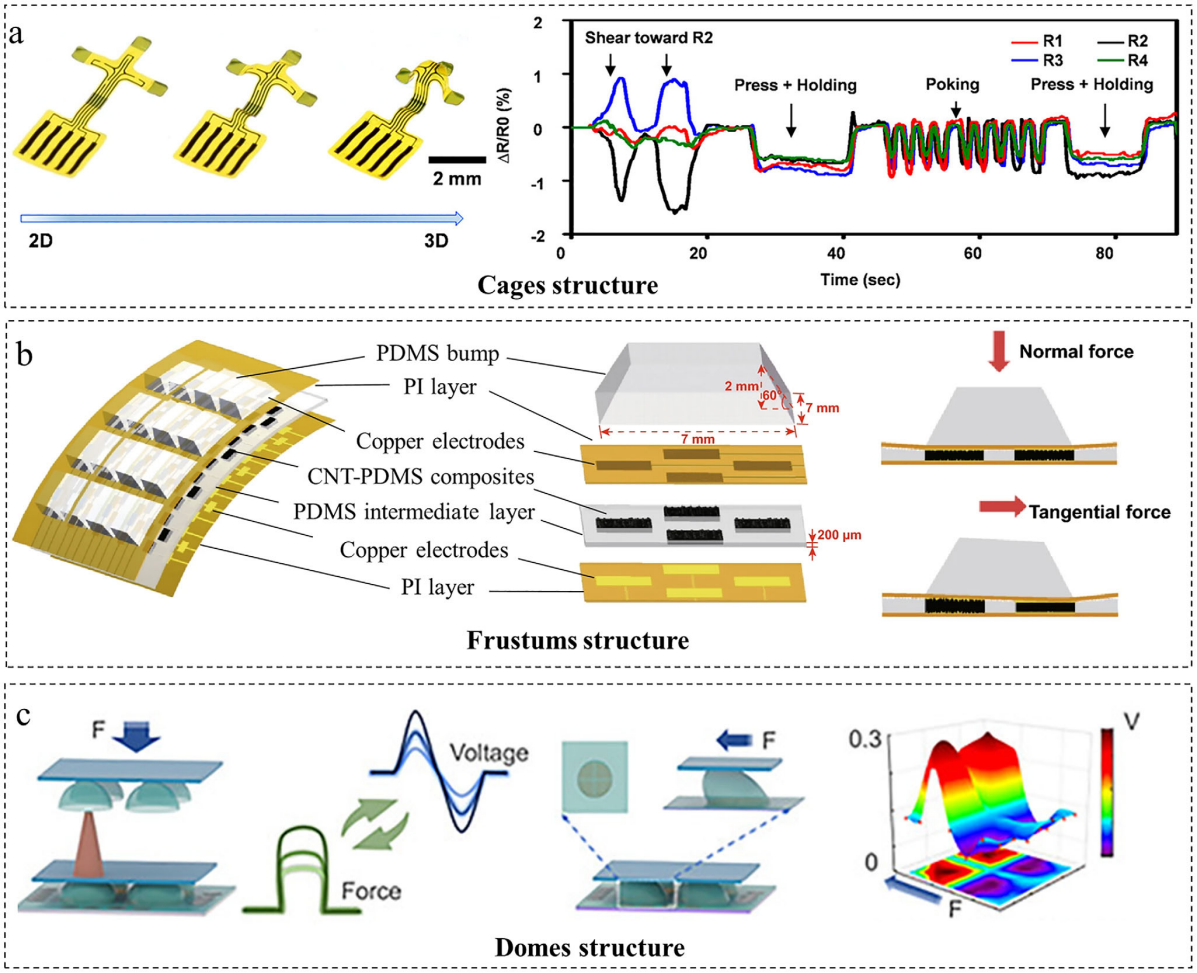
Decoupling through mechanically functional microstructures. (a) Cages structure design and recorded sensing data show responses to shear force and normal pressure. Reproduced with permission [46]. Copyright 2019, American Chemical Society. (b) Frustums structure design and responses to normal/tangential force state. Reproduced with permission [69]. Copyright 2019, Springer Nature. (c) Domes structure design and response of the flexible tactile sensor to the normal/ shear force. Reproduced with permission [75]. Copyright 2024, American Chemical Society.

A direct and effective route to decouple multi‐axis mechanical forces is through standalone microstructured architectures, where controlled and differential bulk deformation inherently isolates mechanical pathways, directly implementing the principle of physical signal isolation. Among various structures, cages, frustums, and domes have proven particularly effective for decoupled sensing of normal pressure and shear forces. As depicted in Figure 6a, Won et al. [46] developed a cage‐inspired 3D architecture with monocrystalline silicon nanomembrane (Si‐NM) piezoresistive elements integrated on a soft substrate, enabling decoupled measurement of pressure, shear forces, bending strain, and temperature. The structure takes the form of a table‐like shape with a planar top surface and four supporting legs fabricated through mechanically guided geometry transformation of a 2D planar precursor. It achieves high linearity (R^2^ >0.99), a response time under 30 ms, and robust durability (>1000 cycles), with calibrated pressure sensitivity of −0.1% kPa^−1^ and shear sensitivity of 0.07% N^−1^ across linear ranges of 0–200 kPa (pressure) and 0–10 N (shear), respectively, providing a scalable and wirelessly integrable solution for flexible electronic skins and wearable devices. Sun et al. [69] developed a 3D contact force detection sensor based on carbon nanotube (CNT)/polydimethylsiloxane (PDMS) nanocomposites. As shown in Figure 6b, through multi‐level architectural design and spatial layout optimization for 3D force decoupling, the sensor integrates four frustums sensing units. Normal forces are calculated from the averaged resistance variation across all units, while tangential forces are determined by differential resistance changes between diagonal units. This design achieves high sensitivity with an ultralow response time of 3.1 ms and precise 3D force vector resolution. As presented in Figure 6c, Han et al. [75] developed a flexible tactile sensor based on an axisymmetric micro‐dome structure, capable of decoupling and simultaneously detecting normal and shear forces (including magnitude and direction). This sensor offers a high directional resolution of 15°, a broad force detection range (0–10 N), and excellent durability, demonstrating its high potential for smart grasping applications in robotics.

Microstructured architectures achieve mechanical force decoupling through geometry‐guided distinct deformation modes, providing direct, hardware‐level signal separation with high linearity and speed. While primarily specialized for force sensing with limited capacity to isolate other signals like temperature, this strategy is compelling for high‐fidelity, multi‐axis tactile applications, such as dexterous robotics and advanced prosthetic haptic feedback.

### 3.2 Planar Designs

Alternatively, decoupling can be achieved through strategic design within 2D planes. This approach relies on localized property contrasts (e.g., wrinkled, kirigami, auxetic) to differentiate signals by engaging independent transduction mechanisms, aligning with the principles of creating deformation‐minimized zones and local stiffening.

**FIGURE 7:**
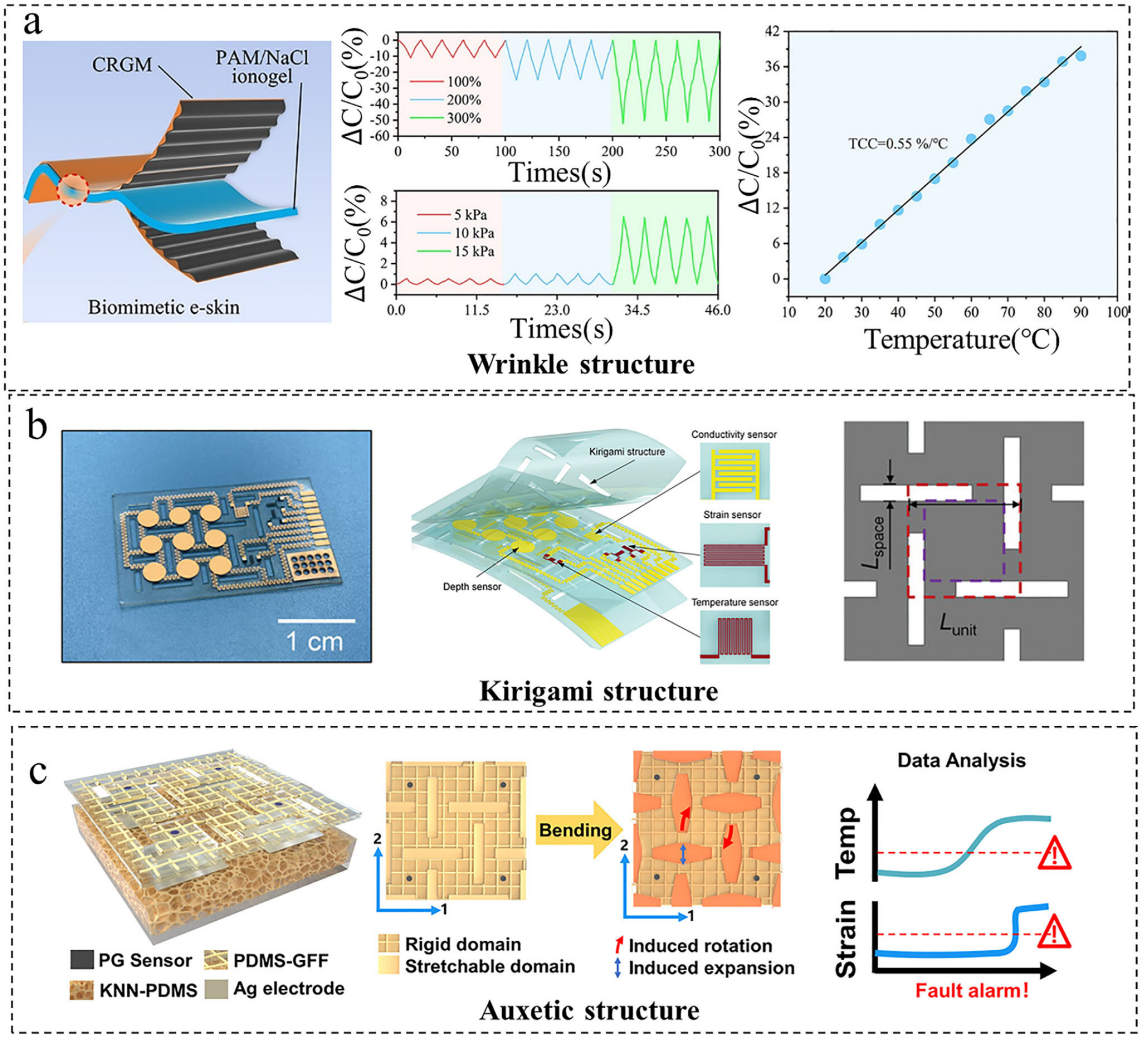
Decoupling through planar designs. (a) Wrinkle structure design and response to pressure and temperature. Reproduced with permission [79]. Copyright 2024, Elsevier lnc. (b) Kirigami structure for decoupled pressure, temperature, and conductivity. Reproduced with permission [80]. Copyright 2023, John Wiley & Sons. (c) Design and strain‐field analysis of an auxetic structure as a decoupling layer for temperature and strain sensing. Reproduced with permission [81]. Copyright 2025, John Wiley & Sons.

Utilizing wrinkled geometric patterns represents one effective approach. As shown in Figure 7a, Bi et al. [79] fabricated a stretchable ionotropic capacitive multimodal sensor by integrating self‐assembled crumpled reduced graphene oxide/multiwalled carbon nanotube films onto natural rubber (NR) through layer‐by‐layer (LBL) electrostatic self‐assembly, employing a polyacrylamide (PAM)/NaCl ionogel as the dielectric layer. The wrinkled electrode facilitates stress sensing by mechanical deformation (tension/compression) that alters the contact area and capacitance between the electrode and the ionogel. Conversely, temperature sensing relies on the thermally responsive ion mobility within the ionogel. These two mechanisms operate independently and exhibit opposite capacitance responses (decreasing under tension and increasing with temperature) as well as distinct response times, thereby achieving signal decoupling without algorithmic processing. The sensor demonstrates high strain sensitivity (gauge factor: 0.27) and linear thermal response (temperature coefficient: 0.55%/°C), making it suitable for bionic electronic skins that mimic natural skin functionality. Zhu et al. [77] constructed independent functional zones for strain and temperature detection through structural design. The strain‐sensing region incorporates localized strain‐concentrating zones, achieving a gauge factor of up to 18.5, a 118% improvement over the baseline value of 8.5. Meanwhile, the temperature‐sensing region features wrinkled structures that enhance the Seebeck coefficient to 122.86 µVK^−1^, representing a 514% increase compared to pristine PEDOT:PSS. This design not only enables structural decoupling of strain and temperature signals over 0%–600% strain but also significantly improves sensitivity, cyclic stability (>1000 cycles), and response speed (0.3 s for strain and 0.75 s for a 0.3 K temperature difference).

Kirigami structures isolate sensing units by channeling strain into island rotation or translation, suppressing crosstalk. He et al. [80] implemented a design with central islands and peripheral hinge bridges on a flexible substrate (Figure 7b), where stretching rotates hinges to dissipate strain, reducing temperature‑sensor crosstalk fivefold. Demonstrated on a robotic dolphin tail for independent underwater monitoring, the approach highlights potential for dynamic wearable sensing.

Auxetic structures provide a distinct decoupling strategy, exemplified by the laser‐patterned rotating‐square auxetic film of Yin et al. [81] (Figure 7c). In this design, rotating rigid units isolate temperature sensing from global strain, while expanding soft units amplify strain for piezoelectric detection, achieving a stable thermal response (25°C–130°C) and high pressure sensitivity (976.97 mV N^−1^) with minimal interference. Similarly, island‑bridge designs offer mechanical decoupling of pressure and shear. Similarly, the island‑bridge design from Guo et al. [47] separates pressure and shear via a central slidable island and peripheral bridges, yielding high shear sensitivity and linear pressure response (0.012 kPa^−1^). Its integrated row/column circuit reduces array ports by ∼83%, enabling scalable use in robotic grippers and wearable motion analysis.

Planar functional structures represent a coherent strategy for achieving hardware‐level decoupling by capitalizing on 2D geometric design and localized material contrasts. A critical challenge is ensuring the long‐term reliability of their micro‐/nanoscale features under cyclic loading. Though promising for bio‐integrated sensing and adaptive robotics, enhancing their environmental robustness remains crucial for translation.

### 3.3 3D Stacking and Integration

**FIGURE 8:**
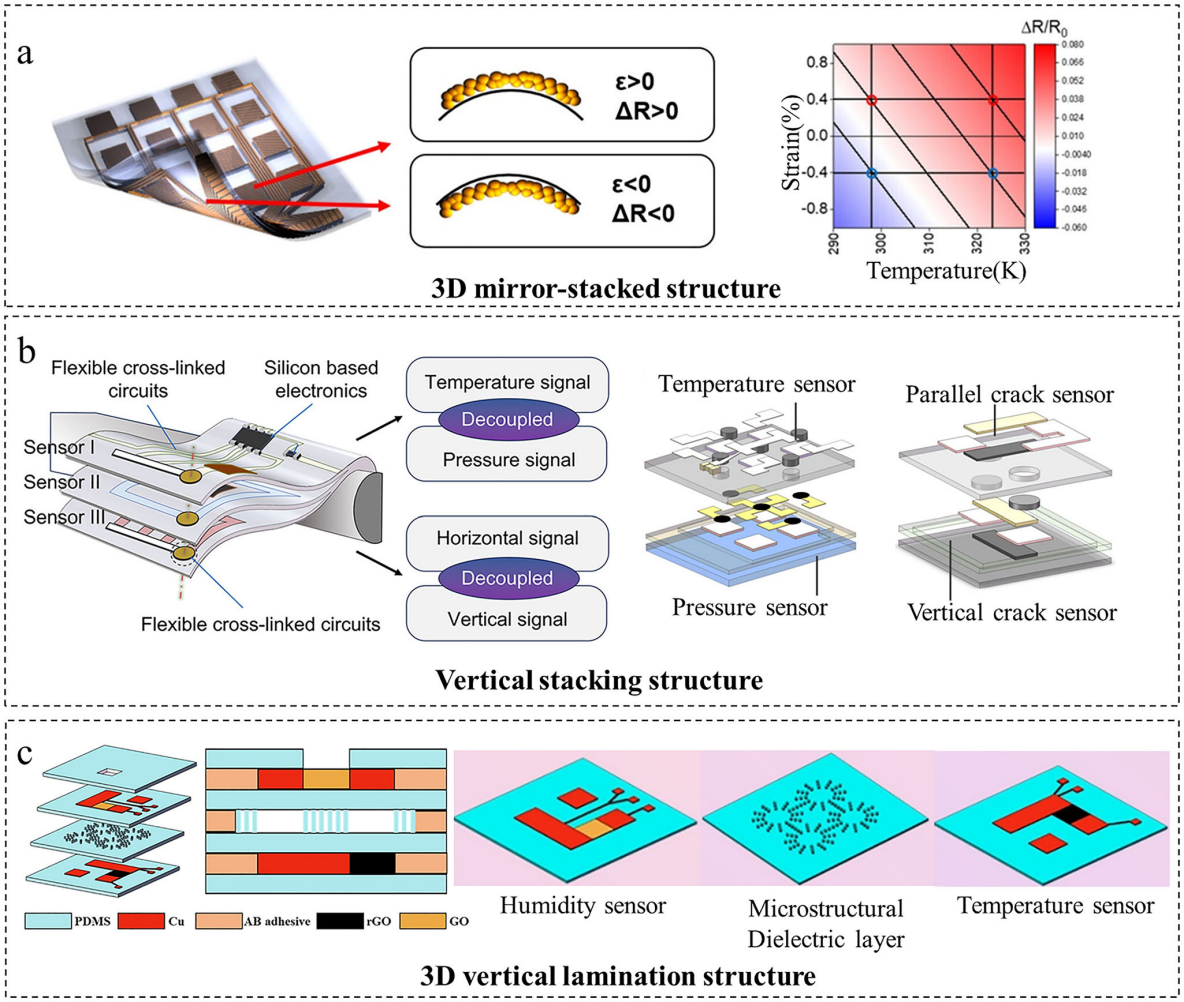
Decoupling through vertical integration architectures. (a) 3D mirror‐stacked design and results of the sensors under different strains and temperatures. Reproduced with permission [82]. Copyright 2019, American Chemical Society. (b) Vertical stacking structure design. Reproduced with permission [35]. Copyright 2024, American Chemical Society. (c) 3D vertical lamination structure design. Reproduced with permission [83]. Copyright 2024, Elsevier lnc.

The 3D structural design enables physical isolation and functional segregation through vertical stacking integration and orthogonal array layout, offering an efficient approach to decoupling multimodal signals. As shown in Figure 8a, Joh et al. [82] achieved simultaneous high‐precision temperature/strain sensing by symmetrically integrating Ag nanocrystal layers on both sides of the neutral mechanical plane via a 3D mirror‐stacked geometry. This design enabled direct strain quantification (±0.8% range, GF = 2.3) while leveraging self‐canceling tensile/compressive effects to maintain ±0.1 K temperature accuracy under 0.4% strain. Combined with a protective Ag_2_O layer from partial oxidation, the sensor extended its temperature range to 673 K (−190 to +400°C) with high thermal sensitivity (TCR = 1.23 × 10^−3^ K^−1^) and rapid response (2.1 s). Zhang et al. [35] developed a fully flexible multimodal sensing system based on a vertical stacking integration strategy, as illustrated in Figure 8b. The design achieved precise temperature‐pressure decoupling (pressure error <±2.1% under 0.8 N load) and multidirectional strain discrimination (GF = 30 perpendicular vs. 6.4 parallel) through orthogonally arranged sensing layers and strain‐insensitive interfaces (volume change <0.5% under deformation). The system also supported room‐temperature adhesive‐free integration (enabling 1336% stretchability) and maintained stable performance under harsh environments (100°C for 1 and 24 h underwater operation). Similarly, as depicted in Figure 8c, Mu et al. [83] developed a crosstalk‐free multimodal sensor via 3D vertical lamination. The device attained hardware‐level decoupling of temperature (sensitivity: 60.64 Ω/°C, R^2^ = 0.998), humidity (ΔC = 72 pF from 15% to 95% RH), and pressure (peak sensitivity: 153.6%/kPa) with very low cross‐sensitivity (<0.6%). Furthermore, it incorporated fully integrated stretchable serpentine circuits for wireless real‐time monitoring of pressure ulcers.

3D integration architectures achieve multimodal decoupling by physically segregating sensors through vertical stacking or orthogonal layouts, enabling isolation at an essentially hardware‐intrinsic level with minimal cross‑talk. However, this performance comes with high fabrication complexity, requiring precise alignment and interfacial bonding of multiple functional layers. Thus, the approach is typically reserved for high‑accuracy systems such as advanced diagnostics and sophisticated human‑machine interfaces.

### 3.4 Fabrication Strategies for Structurally Decoupled Sensors

The practical implementation of sophisticated structural decoupling designs relies heavily on advanced fabrication processes to accurately translate conceptual architectures into functional devices. This transition from design concept to physical implementation poses a significant challenge, making the selection of appropriate fabrication techniques paramount. Key manufacturing methods include spin‐coating [84, 85, 86], transfer printing [87, 88, 89], electrospinning [90, 91], and 3D printing [92, 93], among others are evaluated here for their capabilities in materializing the complex designs discussed earlier, with particular emphasis on their roles in achieving high‐fidelity sensor fabrication and integration.

**FIGURE 9:**
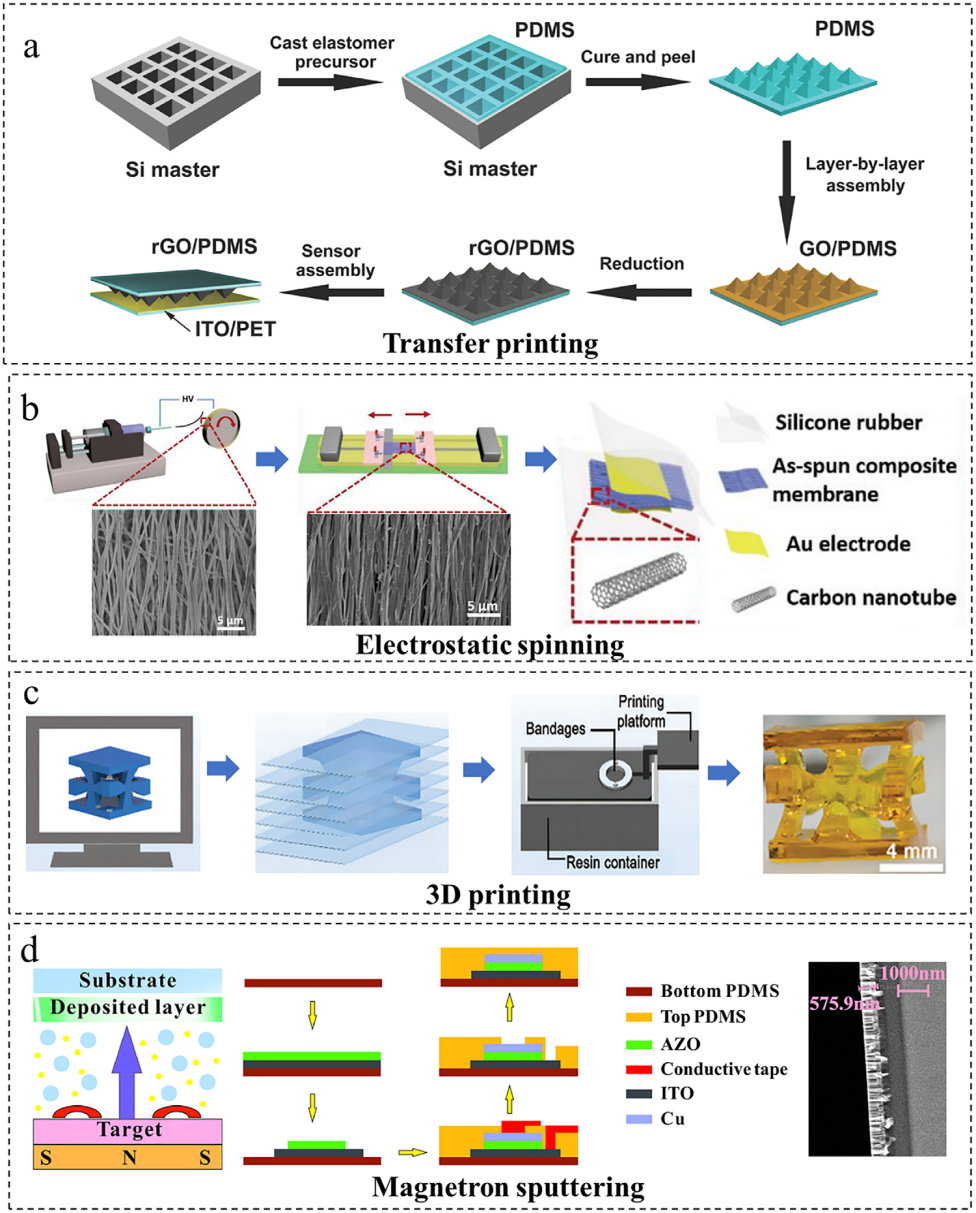
Fabrication of flexible multimodal sensors. (a) Transfer printing. Reproduced with permission [95]. Copyright 2014, WILEY‐VCH GmbH. (b) Electrostatic spinning. Reproduced with permission [98]. Copyright 2018, MDPI. (c) 3D printing. Reproduced with permission [100]. Copyright 2024, WILEY‐VCH GmbH. (d) Magnetron sputtering. Reproduced with permission [105]. Copyright 2024, Elsevier B.V.

Conventional transfer printing employs soft PDMS stamps to transfer nanostructures from rigid growth substrates to flexible ones, enabling the fabrication of sensors [94]. Zhu et al. [95] fabricated a high‐performance flexible resistive tactile sensor using a microstructured molding process (Figure 9a), enabling cost‐effective, simple, and large‐area scalable production. However, the high manufacturing cost and long production cycle of prefabricated silicon molds have restricted their application, which is currently mainly limited to the fabrication of pyramids and frustum structures in structural design.

Electrospinning is a technique that utilizes high‐voltage electrostatic forces to stretch polymer solutions into nanofibers within an electric field [96]. Offering key advantages of low cost and scalable manufacturing, as well as mechanical flexibility and elasticity, it is commonly employed to fabricate wearable sensors like flexible pressure sensors for various applications [97]. As shown in Figure 9b, Wang et al. [98] prepared a composite of PVDF‐TrFE with multi‐walled carbon nanotubes (MWCNTs) using the electrostatic spinning process. The resulting composite film demonstrates excellent piezoelectric properties and high sensitivity, enabling the detection of minute physiological signals.

3D printing provides significant advantages, including rapid manufacturing, cost‐effectiveness, and high material utilization efficiency [99]. This capability facilitates the design and fabrication of complex multimodal sensor architectures while substantially streamlining manufacturing processes. As presented in Figure 9c, Jin et al. [100] fabricated a 3D bio‐inspired kapok structure (3DBIKS) dielectric layer using 3D printing technology, creating a high‐performance flexible capacitive pressure sensor. This design simultaneously achieved high sensitivity (≈2.38 kPa^−^^1^ in 0–10 kPa), an exceptionally broad detection range (up to 734 kPa), rapid response/recovery (23 ms/40 ms), low detection limit (20 Pa), high‐pressure resolution (0.4% at 500 kPa), and low hysteresis (8.4%), thereby resolving the fundamental trade‐off between sensitivity and detection range in flexible sensors.

In addition to the techniques described above, several other methods, such as lithography [101, 102] and physical/chemical vapor deposition (PVD/CVD) [103, 104], were also commonly used for fabricating sensors. Lithography utilizes photoresists to create high‐resolution patterns and has found extensive industrial and laboratory applications in device fabrication. As shown in Figure 9d, PVD/CVD techniques such as magnetron sputtering [105], which deposit thin films on substrates under specialized conditions (e.g., high temperature/vacuum), are ideal for high‐performance sensors yet face material and scalability constraints.

Structural decoupling achieves hardware‐level signal separation by mechanically isolating cross‐talk pathways through integrated physical segregation, strategic element placement, and localized stiffening. These approaches, which include microstructured architectures, planar designs, and 3D integration, enable the direct separation of multimodal signals (e.g., pressure, shear, strain, temperature) with high linearity. Despite these advanced structural and manufacturing strategies, certain forms of signal interference, such as residual thermo‐mechanical crosstalk may still persist. These limitations motivate the incorporation of AI‐based decoupling methods, which will be discussed in the following section.

## 4 AI‐Based Signal Decoupling

While advances in multifunctional sensing materials and sophisticated structural designs facilitate the simultaneous detection of diverse stimuli, they inherently introduce complex signal coupling and cross‐sensitivity among the measured physical variables. Traditional electronic decoupling circuits, which rely predominantly on linear models or empirical calibration routines, are fundamentally inadequate to characterize the intricate, nonlinear interactions arising from these advanced material systems and structural architectures. To address this limitation, AI‐driven computational approaches have been introduced, harnessing machine learning and deep learning to construct sophisticated nonlinear mappings for signal decoupling. These include shallow models (e.g., support vector machines and backpropagation neural networks), deep neural networks (DNNs, CNNs), multi‐algorithm fusion strategies that combine data preprocessing with neural networks, and hardware‐software co‐design decoupling methods. Collectively, these techniques enable highly accurate decoupling in multimodal sensing systems.

### 4.1 Shallow Models

**FIGURE 10:**
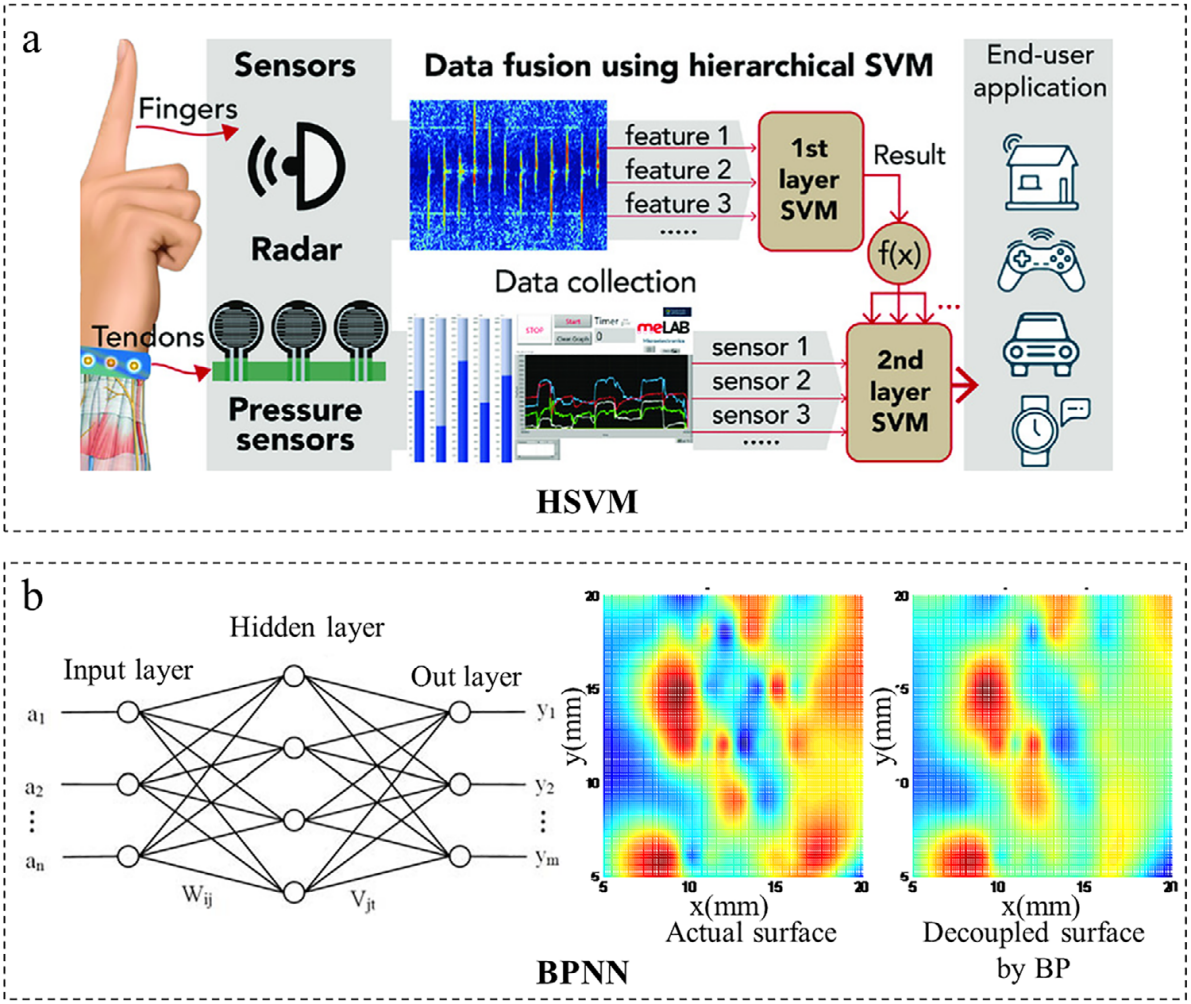
Decoupling through shallow models. (a) Conceptual Diagram Illustrating Multi‐modal Gesture‐Sensing Data Fusion via HSVM. Reproduced with permission [38]. Copyright 2019, WILEY‐VCH GmbH. (b) The model of BPNN and the fitting surface for the decoupling results. Reproduced under terms of the CC‐BY Creative Commons Attribution 4.0 International License (http://creativecommons.org/licenses/by/4.0/) [48]. Copyright 2013, Feilu Wang, published by IFSA.

Shallow models, such as Support Vector Machines (SVM) and Backpropagation Neural Networks (BPNN), serve as effective computational tools for decoupling multimodal sensor signals, particularly in scenarios with limited data complexity or constrained hardware resources. For instance, Liang et al. [38] employed a Hierarchical SVM (HSVM) architecture to fuse heterogeneous data from radar and pressure sensors (Figure 10a), significantly improving gesture recognition accuracy from 76.7% (radar alone) and 69.0% (pressure alone) to 92.5% through optimized feature‐level fusion. Similarly, compact single‐hidden‐layer BPNNs have demonstrated strong decoupling performance with minimal computational overhead. As shown in Figure 10b, Wang et al. [48] proposed a decoupling method for a flexible tactile sensor array using an optimized Back‐Propagation Neural Network (BPNN). By feeding multi‐channel resistance signals into a single‐hidden‐layer BPNN with 12 nodes, the method successfully decoupled 3D deformation coordinates from coupled sensor responses. Experimental results demonstrated 98.59% prediction accuracy (Z‐axis error 1.41%) under optimal array scale, validated through 10‐fold cross‐validation. In a separate study, Wang et al. [106] developed a 3D force sensor based on a Back‐Propagation Neural Network (BPNN), which successfully decoupled force directions (Fx, Fy, Fz) by feeding four‐channel capacitive signals into a single‐hidden‐layer BPNN model, achieving 98.1% prediction accuracy after 2000 iterations.

Shallow models like SVM and BPNN provide a practical, low‑cost approach to signal decoupling in scenarios where computational resources are constrained. Their straightforward implementation suits embedded or portable systems well. However, limited representational capacity restricts their effectiveness for highly nonlinear interactions or large‑scale, high‑dimensional data. Thus, they are best applied to decouple moderately complex signals when training data is scarce or real‑time operation is essential.

### 4.2 Deep Neural Networks

**FIGURE 11:**
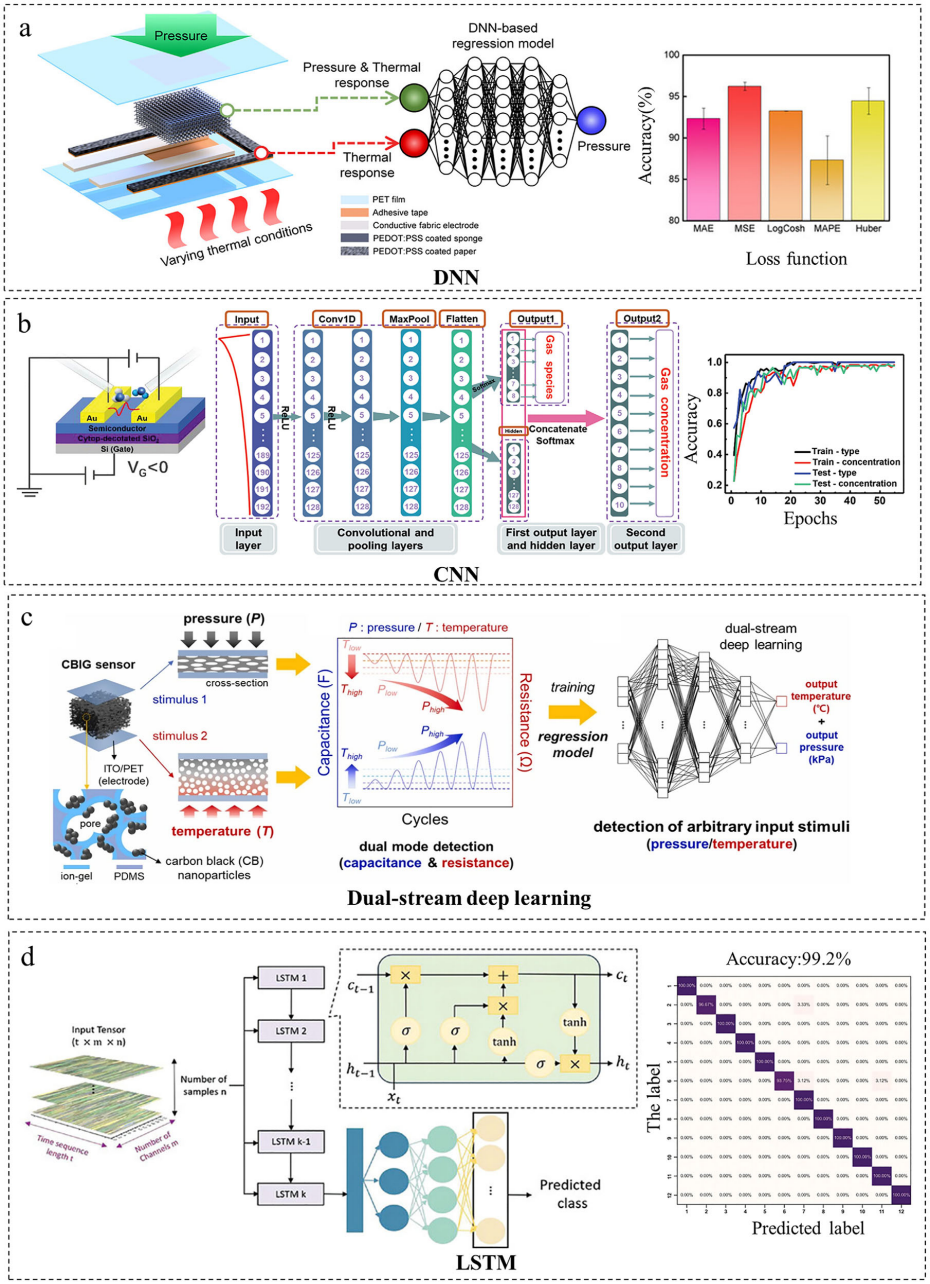
Decoupling through deep neural networks. (a) Schematic and conceptual diagram of DNN based thermal decoupling system. Reproduced with permission [49]. Copyright 2023, WILEY‐VCH GmbH. (b) CNN Process Model for Gas Type and Concentration Identification. Reproduced with permission [108]. Copyright 2024, WILEY‐VCH GmbH. (c) Conceptual design, device structure, and manufacturing process of dual‐stream deep learning integrated multimodal sensors. Reproduced with permission [109]. Copyright 2024, Elsever Ltd. (d) Structural diagram of LSTM model. Reproduced with permission [110]. Copyright 2024, WILEY‐VCH GmbH.

However, such shallow models are limited in their ability to process large‐scale datasets and capture highly intricate variable relationships, prompting the adoption of deeper neural networks [107]. Bang et al. [49] tackled the decoupling of temperature interference from pressure signals using a deep neural network (DNN) regression model (Figure 11a). Their approach featured a dual‐sensing configuration comprising a cellulose paper‐based thermally responsive unit (temperature‐only) and a porous sponge‐based unit (pressure and temperature). By training on 8016 data points with min‐max normalization and employing a four‐layer fully connected DNN with Dropout regularization, they achieved a pressure prediction accuracy of 96.23%. The DNN effectively modeled the complex nonlinear coupling between temperature and pressure, outperforming traditional methods based on piecewise calibration or linear assumptions. While DNN regression models excel at capturing complex interactions in continuous data, their increasing depth and width can lead to the attenuation of early‐stage feature information. This has motivated the use of alternative architectures such as Convolutional Neural Networks (CNNs), which are particularly effective at preserving spatial and local information. Wu et al. [108] simulated the excitation‐inhibition balance mechanism of the human olfactory system using a convolutional neural network (CNN) shown in Figure 11b. Their bio‐inspired synaptic device generates complex signals by modulating gas pulse parameters (intensity, duration). Compared to traditional Fourier transform or statistical methods, the CNN employs sliding convolutional kernels to perform locally weighted summation of time‐domain current signals, preserving spatial information while integrating neuron‐level signals. This achieves 97% classification accuracy for eight gases (e.g., ethyl butyrate, hydrogen sulfide) with significantly enhanced sensitivity to concentration variations.

Separately, to address the challenge of decoupling physically intermixed signals in multimodal sensing, Kim et al. [109] proposed a dual‐stream deep learning framework (Figure 11c) comprising one regression branch for pressure signals and another for temperature signals, with a feature fusion layer for joint prediction. The model achieved mean absolute percentage errors (MAPE) of 1.58% for pressure and 2.37% for temperature, demonstrating high reliability in synchronous monitoring of grasping force and surface temperature for intelligent prosthetics. For complex temporal modeling, Lee et al. [110] designed a multilayered flexible sensor integrating triboelectric (pressure), piezoresistive (hardness), and curvature‐sensing components. As shown in Figure 11d, their architecture processes multimodal time‐series data through hierarchical LSTM networks to capture long‐range dependencies, augmented by attention mechanisms for adaptive feature fusion. This approach achieved 99.2% recognition accuracy across 12 object categories in dynamic grasping scenarios.

DNNs and CNNs are well‐suited for complex decoupling tasks due to their ability to learn intricate, nonlinear mappings from large datasets, with CNNs particularly effective at extracting spatiotemporal features. Key drawbacks include their substantial demand for labeled data and computational resources, alongside a risk of overfitting. These models are best applied to high‑dimensional sensing systems, such as tactile arrays or multimodal time‑series data, where signal relationships are highly complex and sufficient training data exists.

### 4.3 Multi‐Algorithm Fusion or Hardware‐Software Co‐Design

**FIGURE 12:**
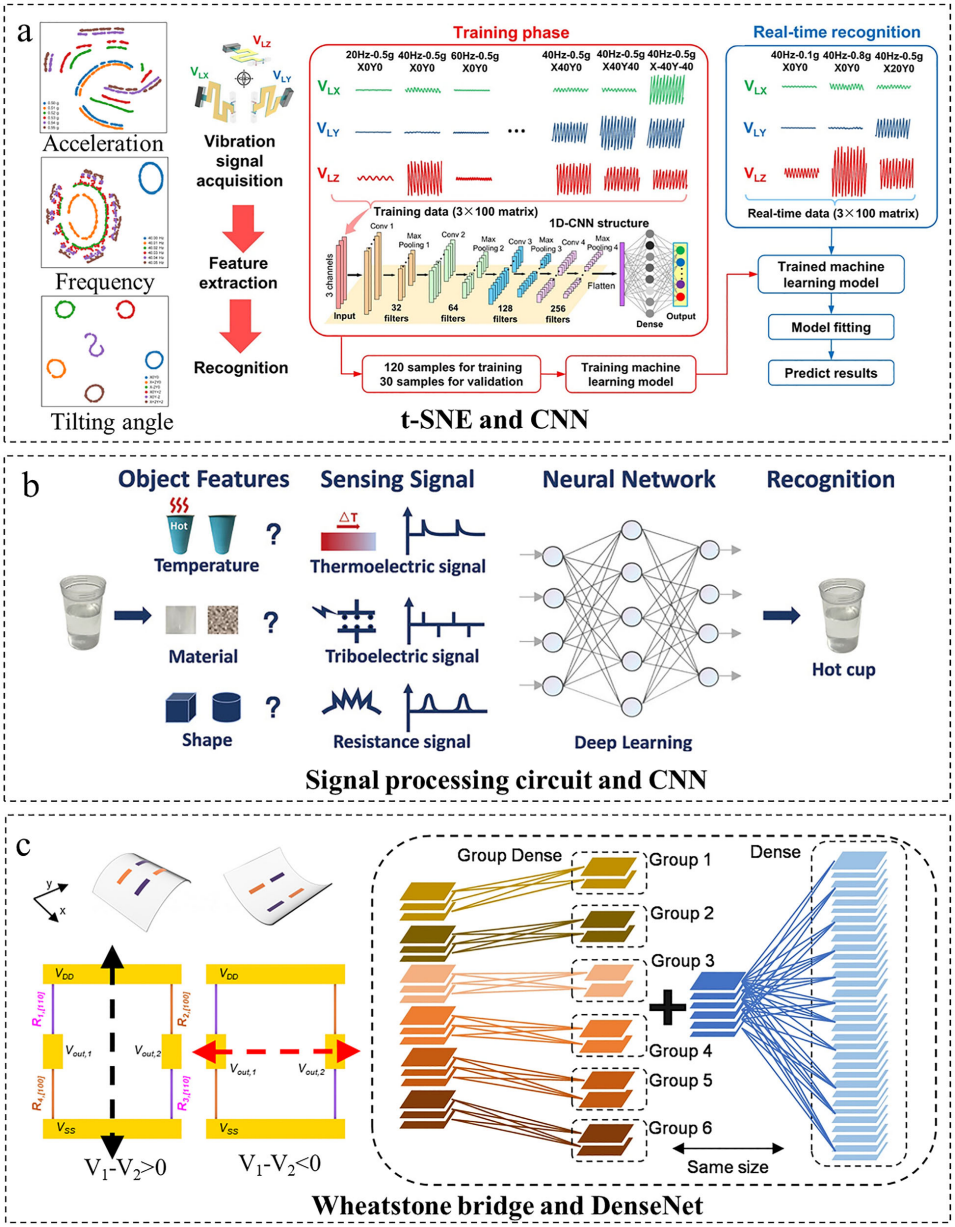
Decoupling through multi‐algorithm fusion and hardware‐software co‐design. (a) t‐SNE visualization and CNN architecture for multimodal vibration sensing, including acceleration, frequency, and tilting angle. Reproduced with permission [50]. Copyright 2023, American Chemical Society. (b) System architecture integrating the signal processing circuit and CNN for object recognition. Reproduced with permission [112]. Copyright 2024, WILEY‐VCH GmbH. (c) Co‐design of a Wheatstone bridge sensor and group convolution‐enhanced DenseNet for tactile classification. Reproduced with permission [113]. Copyright 2022, Springer Nature.

Beyond purely deep learning‐based methods, hybrid algorithmic strategies have emerged as a promising alternative for complex multi‐parameter decoupling tasks. Ding et al. [111] pioneered a dual‐neural‐network decoupling framework for flexible 3D tactile sensors, integrating BPNN for deformation analysis (single hidden layer with 12 nodes, optimized via 10‐fold cross‐validation) and direct resistance‐to‐force RBFNN (Gaussian kernel with spread = 1). This hybrid approach achieved 0.11% error in Fy‐axis decoupling and maintained <10% Fz‐axis error under 10% white Gaussian noise, enabling robust force sensing for industrial robotic manipulation in noisy environments. Huang et al. [50] exemplified this by integrating a self‐powered triaxial piezoelectric sensor (TPS) with complementary deep learning techniques, specifically combining t‐SNE for feature‐space analysis and CNN for pattern recognition in their iCUPE system (Figure 12a), achieving 98%–100% accuracy in multifunctional vibration sensing across acceleration, frequency, and tilt within industrial AIoT environments.

Through co‐design of hardware and neural algorithms, researchers have achieved precise multi‐modal perception by leveraging inherent signal decoupling. As shown in Figure 12b, Wang et al. [112] developed a multifunctional sensor based on graphene‐coated textiles and ZnO/PDMS composite films. This sensor achieves tri‐modal perception of strain, material types, and temperature through three decoupled physical mechanisms: piezoresistive (strain), triboelectric (material), and thermoelectric (temperature) effects. By integrating an embedded signal processing circuit with a convolutional neural network (CNN) for end‐to‐end fusion of raw multi‐modal signals, the system attained 98.5% accuracy in identifying five categories of objects with distinct materials (including metals, plastics, and wood) under real‐world grasping scenarios. To address the classification of complex mechanical stimuli, So et al. [113] developed a customized deep learning model shown in Figure 12c integrated with a flexible piezoresistive sensing system configured in a Wheatstone bridge, utilizing ultrathin single‐crystalline silicon nanomembranes (Si‐NMs) with distinct crystallographic orientations to generate differentiated strain response. By incorporating a group convolution design within the DenseNet architecture, the model independently learns nonlinear relationships between mechanical stimuli (such as concave bending along the x‐axis and convex bending along the y‐axis) and substrate hardness from 180 datasets, achieving prediction accuracy surpassing conventional models. Furthermore, leveraging continuous wavelet transform (CWT) and EfficientNet, the system converts time‐domain signals into image features, enabling high‐precision classification of objects like earbuds and cotton swabs with a validation accuracy of 96.9%.

This paradigm of end‐to‐end co‐optimization, from sensing physics to information decoding, often yields significant improvements in system‐level performance and efficiency. Its development, however, relies on deep interdisciplinary integration and may compromise generalizability and post‐deployment flexibility. Consequently, it is most effective in specialized applications with stringent requirements on integration, power consumption, and real‐time processing, such as advanced wearable devices and robotic perception systems.

AI‐based signal decoupling methods, such as machine learning and deep learning algorithms resolve the inherent signal entanglement stemming from multifunctional material integrations and structure design. Shallow models favor efficiency over capacity, deep networks deliver high accuracy at the cost of data and computation, hybrid approaches enhance robustness through added complexity, and co‑design optimizes integration while limiting flexibility. The optimal choice thus depends on specific application constraints such as signal complexity, data availability, hardware resources, and real‑time needs. By establishing task‐optimized nonlinear mappings, these approaches achieve exceptional decoupling precision and multimodal fusion capabilities, thereby establishing sophisticated functionalities approaching human perceptual intelligence.

## 5 Application of Flexible Multimodal Sensors

Flexible multimodal sensors enable the synchronous perception of diverse physical/chemical signals, including pressure, strain, temperature, humidity, light, and biochemical molecules. However, their development is hindered by inherent signal coupling and crosstalk. To address this, the preceding sections have established a framework of core decoupling strategies based on material design, structural design, and algorithmic approaches. This section reviews representative applications in environmental monitoring, health monitoring, human‐machine interaction, and intelligent robotics, with a dedicated focus on analyzing the core decoupling strategy employed in each case. The analysis aims to elucidate how these methodological innovations drive functional breakthroughs and to clarify the key obstacles toward practical utility.

### 5.1 Environmental Monitoring

Multimodal sensing technology is transforming environmental monitoring through the integrated acquisition of diverse ecological parameters, enabling a more holistic understanding of environmental dynamics and their health implications. By simultaneously capturing multiple sensing modalities, this approach significantly improves data accuracy, temporal resolution, and comprehensiveness, thereby providing critical tools for predictive risk management and advanced research on environment‐health correlations across various exposure scenarios.

**FIGURE 13:**
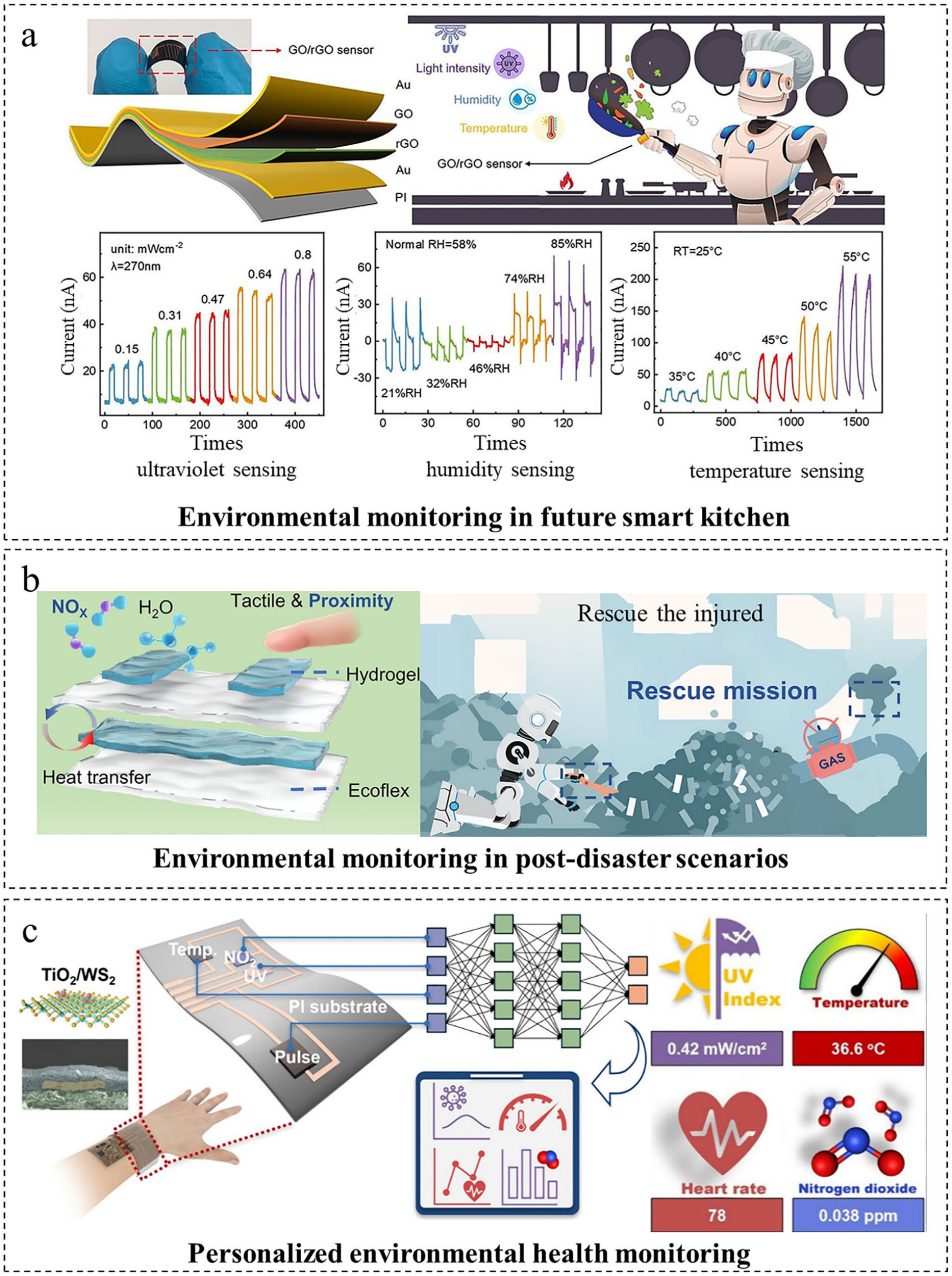
Applications of flexible multimodal sensors in environmental monitoring. (a) Multimodal e‐skin system for environmental monitoring in post‐disaster scenarios, detecting toxic gases (NO_2_), temperature, humidity, pressure, and proximity. Reproduced with permission [114]. Copyright 2024, Springer Nature. (b) Application of the GO/rGO bilayer film device for UV, humidity, and temperature monitoring in future smart kitchen robots. Reproduced with permission [115]. Copyright 2025, Chemical Engineering Journal. (c) A wearable multimodal sensor for personalized environmental health monitoring, simultaneously tracking exposure to NO_2_ and UV radiation. Reproduced with permission [116]. Copyright 2025, American Chemical Society.

As illustrated in Figure 13a, Li et al. [114] developed a multimodal e‐skin capable of precise environmental monitoring and risk warning through the co‐design of deep neural networks (DNN) with multi‐physics sensing units to algorithmically fuse and decouple signals. The system demonstrated high selectivity to NO_2_, with a detection error of <±8% and a detection limit of 11.1 ppb under 85% humidity across a temperature range of −10°C to 60°C, while exhibiting a sensitivity of 254%   ppb^−1^. The system triggers a three‐level alarm within 50 ms when the NO_2_ concentration exceeds the safety threshold (>50 ppb). This multilayered and stretchable platform, integrated with a custom wireless alarm circuit and Bluetooth transmission, enables real‐time environmental awareness for rescue robots in post‐earthquake scenarios.

Besides, as presented in Figure 13b, Wang et al. [115] developed a GO/rGO bilayer film sensor whose operation relies on a material‐centric proton migration mechanism, enabling self‐powered, synchronous monitoring and intrinsic decoupling of UV light sensitivity (58.2 mA/mW·cm^2^), humidity response (+11.6 mA/ΔRH during increase and −0.68 mA/ΔRH during decrease), and temperature sensitivity (2.63 nA/°C in low‐temperature range; 12.4 nA/°C in high‐temperature range) via a proton migration mechanism. This breakthrough in self‐powered multisensory monitoring opens new possibilities for applications in smart kitchen environments and complex ecological assessment.

Beyond targeted gas monitoring, Jia et al. [116] invented a wearable multimodal sensing system using a titanium dioxide/tungsten disulfide (TiO_2_/WS_2_) heterojunction to synchronously monitor environmental nitrogen dioxide (NO_2_) and UV radiation intensity. As presented in Figure 13c, this platform exemplifies a synergistic decoupling approach, combining heterojunction material engineering with monolithic structural integration on flexible printed circuit boards (FPCB) with Bluetooth transmission, enables wrist‐worn health‐environment monitoring. The system achieves high‐precision synchronous detection of four parameters: NO_2_ (14.4 ppb detection limit), UV intensity (0.024 mW/cm^2^), body temperature (0.22% sensitivity per °C), and pulse (180 ms response time), providing a reliable tool for personalized studies of health‐environment exposure correlations. Misra et al. [117] developed a self‐powered multimodal sensing platform leveraging nanomaterials and flexible integration processes. This system achieved ozone detection at 20 ppb (parts per billion), enabling correlation with respiratory symptoms (e.g., wheezing), and tracked toluene exposure against the OSHA permissible limit (200 ppm). These capabilities establish a technological foundation for investigating health‐environment interactions, particularly in occupational safety applications. Advancing toward truly autonomous and persistent environmental monitoring requires innovations not only in sensing but also in sustainable power. Exemplifying this, Guo et al. [118] developed a high‐performance, leaf‐based energy harvester that decouples high power output from long‐term stability, offering a promising avenue for future self‐powered environmental sensing systems.

Multimodal sensing technologies significantly advance environmental monitoring by integrating the detection of diverse ecological parameters, thereby improving the accuracy, responsiveness, and comprehensiveness of acquired data. Such systems not only facilitate real‐time risk alerts but also support in‐depth research on the correlations between environmental exposure and human health, offering robust tools for both preventive interventions and scientific exploration.

### 5.2 Physiological Health Monitoring

Recent advances in flexible multimodal sensing technologies are fundamentally transforming wearable healthcare by enabling comprehensive, non‐invasive monitoring of physiological signals with unprecedented clinical utility. These integrated systems transcend single‐parameter tracking through synergistic data fusion, delivering predictive insights and personalized interventions that redefine patient care paradigms.

**FIGURE 14:**
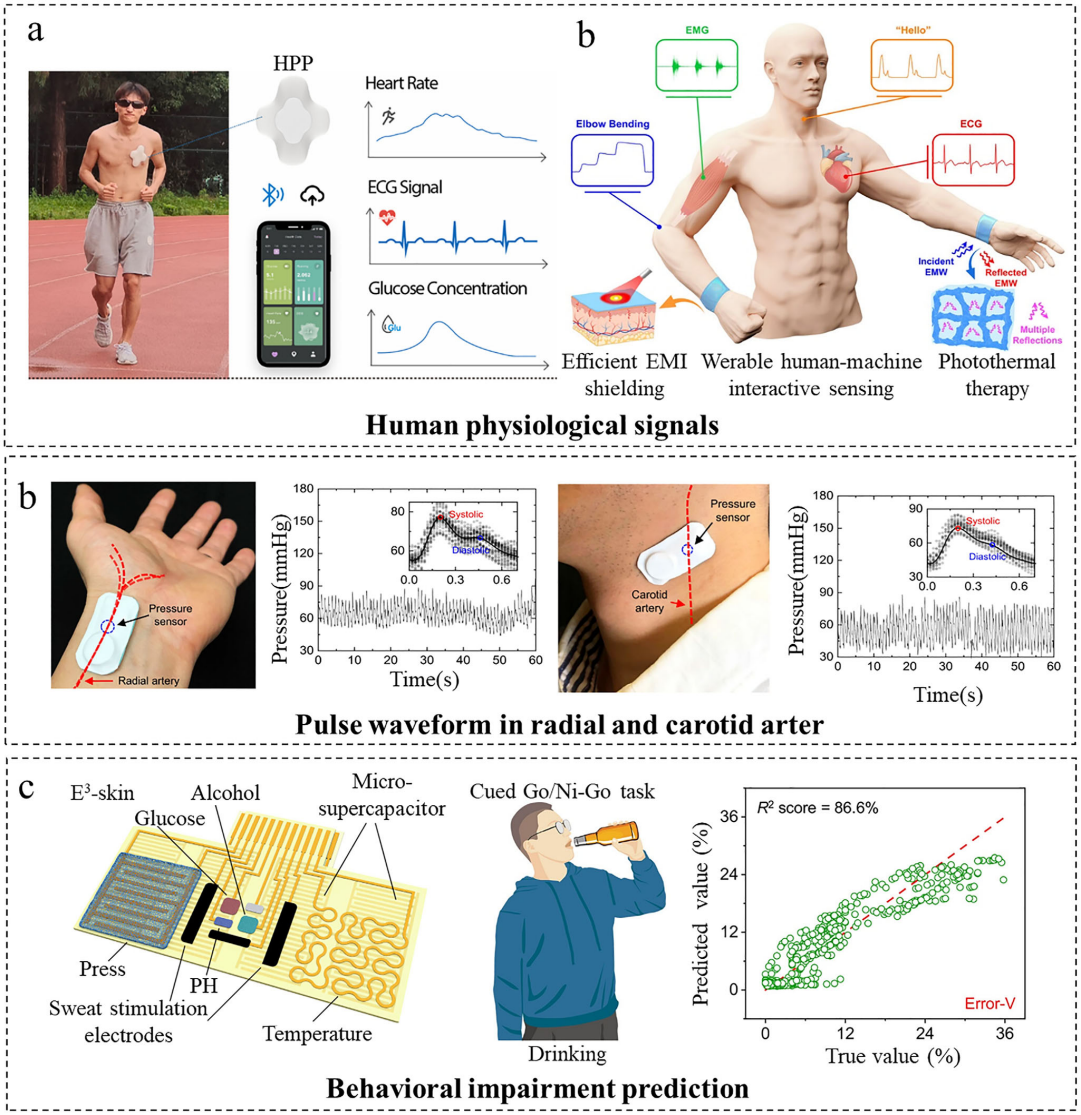
Applications of flexible multimodal sensors in physiological health monitoring. (a) Custom‐developed HPP device for electrophysiological (ECG) and biochemical (glucose) monitoring. Reproduced with permission [119]. Copyright 2021, Elsevier B.V. (b) Flexible self‐healing MXene hydrogel multimodal sensor for physiological signal detection. Reproduced with permission [121]. Copyright 2024, John Wiley & Sons. (c) Wireless pressure sensing system for monitoring pulse waveform in radial and carotid arteries. Reproduced with permission [122]. Copyright 2024, Springer Nature. (d) Behavioral impairment prediction via fully integrated e^3^‐skin platform. Reproduced with permission [123]. Copyright 2023, The American Association for the Advancement of Science.

The convergence of electrophysiological and biochemical sensing represents a significant frontier in flexible wearable monitoring. Li et al. [119] developed an integrated conductive hydrogel‐paper patch (HPP) for simultaneous sensing of electrophysiological (ECG) and biochemical (glucose) signals in sweat. As illustrated in Figure 14a, this flexible and disposable platform, fabricated using wax printing and PEDOT:PSS hydrogel self‐assembly on paper fiber, enables real‐time chest‐worn health monitoring during exercise. The system achieves synchronous detection of two key physiological parameters: ECG (with impedance lower than commercial electrodes in low‐frequency regions) and sweat glucose (with a sensitivity of 325.99 µA mM^−^^1^ cm^−^^2^ and a detection limit of 10.3 µm), providing a low‐cost, conformable solution for comprehensive fitness monitoring and athlete fatigue assessment. Complementing this approach, several research groups have developed specialized sensing systems targeting specific healthcare applications. Moon et al. [120] developed a wearable multimodal wireless sensing system that integrates an acoustic sensor (piezoelectric) and an electrocardiogram (ECG) sensor for real‐time monitoring of respiratory activities and detection of abnormal breathing patterns such as shallow breathing and coughing.

Similarly, Zhang et al. [121] have developed a flexible self‐healing MXene hydrogel multimodal sensor, capable of simultaneously detecting human physiological signals (e.g., ECG, EMG, joint movements), and employs machine learning algorithms to decouple and recognize gestures with 99.5% accuracy through machine learning, and integrating photothermal therapy with electromagnetic shielding (48.95 dB) to achieve a closed‐loop diagnosis and treatment system (Figure 14b). For cardiovascular monitoring, Park et al. [122] developed a flexible 3D multimodal pressure sensor shown in Figure 14c that leverages a Wheatstone bridge structural configuration for mechanical decoupling of decouples normal pressure from shear stress while eliminating environmental interference via autonomous temperature compensation, enabling real‐time wireless monitoring of cardiovascular activities (e.g., radial/carotid arterial pulse waveforms) with clinical‐grade accuracy (≤1.2 mmHg mean deviation in mean arterial pressure during Valsalva maneuver validation against reference systems), thus providing a portable clinical‐grade solution for precision home‐based management of hypertension and other chronic conditions.

Multimodal sensors further provide comprehensive risk detection and prediction through non‐invasive, continuous monitoring, facilitating chronic disease management and early warning systems for high‐risk conditions. Employing a novel semi‐solid extrusion 3D printing technique, Song et al. [123] fabricate a fully integrated, epifluidic electronic skin (e^3^‐skin) for advanced health monitoring (Figure 14d). The device constitutes a closed‐loop system that combines sweat biosensing (glucose, alcohol, pH) and vital sign tracking with iontophoretic sweat stimulation and energy harvesting. Marrying this multimodal data with machine learning enabled accurate (>90%) prediction of alcohol‐induced behavioral impairment, showcasing a scalable manufacturing platform for autonomous, personalized wearable systems. Kuldeep et al. [124] developed a wearable hybrid multimodal patch integrating biophysical and biochemical sensors for synchronous monitoring of critical human vital signs (such as ECG, blood pressure, oxygen saturation) and biomarkers (including glucose, lactate, cortisol), enabling integrated health management for conditions like diabetes, cardiovascular diseases, and sepsis. Oh et al. [125] developed a flexible multimodal sensing system for clinical application, which is attached to high‐risk pressure ulcer areas such as the sacrum and heels to continuously monitor pressure distribution and localized temperature increases. Long‐term monitoring of paraplegic patients demonstrated that this system provides early warnings by algorithmically analyzing pressure‐temperature time‐series data to decouple correlated risk factors, offering alerts up to 2 h in advance.

These multimodal systems transform wearable healthcare by converting correlated sensor data into predictive health foresight, enabling early interventions that shift chronic disease management from reactive treatment to proactive prevention.

### 5.3 Human‐Machine Interaction

Human‐machine interaction (HMI) is undergoing a paradigm shift through multimodal sensing technologies, which enable closed‐loop integration of user inputs, system responses, and environmental contexts across diverse applications. This technological evolution is particularly transformative in object recognition, bionic skin development, and immersive virtual systems, where multimodal approaches overcome fundamental limitations of unimodal interfaces.

**FIGURE 15:**
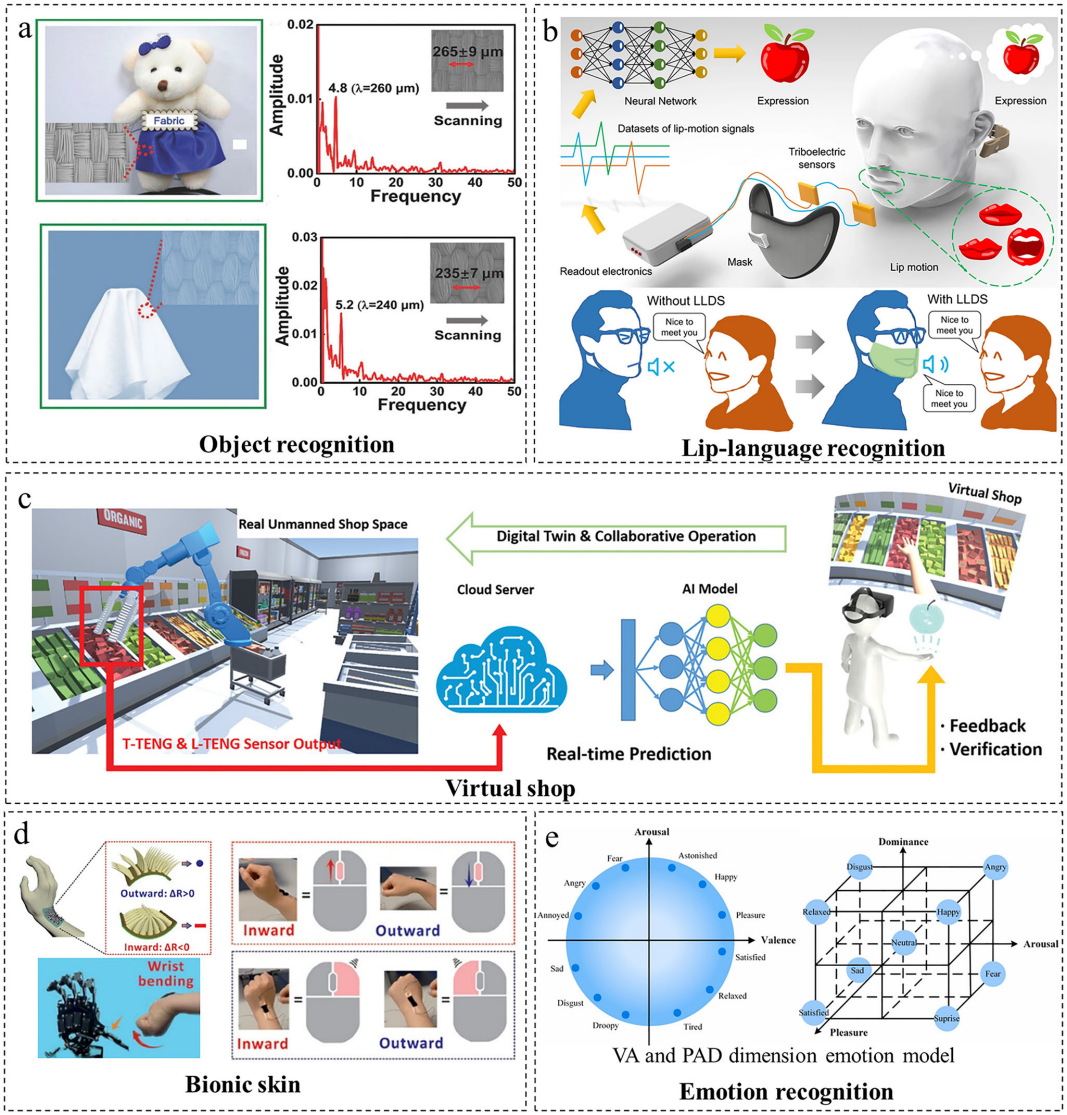
Applications of flexible multimodal sensors in human‐machine interaction. (a) The identification of different silk fabrics and Braille characters reading. Reproduced with permission [126]. Copyright 2018, WILEY‐VCH GmbH. (b) The applications for lip‐language decoding in communication for people lacking a voice. Reproduced with permission [127]. Copyright 2022, Springer Nature. (c) The digital‐twin‐based virtual shop and the 28 objects with different shapes and sizes to be identified by the system. Reproduced with permission [129]. Copyright 2021, WILEY‐VCH GmbH. (d) Schematic diagram of adhesive E‐skin attached on the human wrist. Reproduced with permission [133]. Copyright 2024, WILEY‐VCH GmbH. (e) Two typical dimension emotion models. Reproduced with permission [135]. Copyright 2023, Elsevier B.V.

Object recognition facilitates applications in areas including Braille reading and garbage sorting through the identification of object shapes, materials, and textures. As presented in Figure 15a, Cao et al. [126] proposed a bionic electronic fingerprint‐inspired flexible tactile sensor that achieves high‐precision surface micro texture recognition through a multimodal sensing mechanism combining pressure and shear force detection. In texture discrimination validation experiments, this sensor successfully demonstrated accurate identification of diverse fabric textures and Braille characters reading. As presented in Figure 15b, Lu et al. [127] proposed a dilated recurrent neural network model based on prototype learning that achieves high‐accuracy lip‐language recognition through a deep learning framework combining feature extraction and prototype matching. In classification experiments on 20 categories of lip‐motion signals, this model successfully demonstrated a test accuracy of 94.5%, significantly outperforming traditional softmax‐based methods, especially under small‐sample conditions. Beyond visual cues, tactile sensing provides another powerful pathway for recognition and communication. Utilizing a material‐level decoupling strategy, Zhou et al. [128] constructed a hydrogen‐bond network within a thermoplastic polyurethane matrix to firmly anchor silver nanowires and ionic liquids. This material design enabled the fabrication of an ultrasensitive and robust flexible pressure sensor, which was integrated into a smart glove for intelligent sign language recognition. The system achieved a high recognition accuracy of 96.8% for 24 letters with a rapid response time of less than 0.1 s, demonstrating a practical solution for assistive communication.

Besides, Sun et al. [129] invented a multi‐modal self‐powered sensing system integrating a triboelectric bending sensor (L‐TENG), a tactile sensor (T‐TENG), and a pyroelectric temperature sensor (PVDF) for real‐time detection of shape, size, and temperature distribution information of grasped objects in virtual shop applications, shown in Figure 15c. The system employs machine learning as the core strategy to fuse and decouple the multi‐modal data, automatically recognizing 28 distinct object shapes with 97.1% accuracy, while digital twin technology delivers an immersive shopping experience. Zhong et al. [130] invented a high‐density active‐matrix tactile sensor based on intrinsically stretchable transistor arrays, enabling detection of submillimeter‐scale object shapes, sizes, orientations, and braille dot patterns with resolution surpassing human fingertip perception by over tenfold, while simultaneously supporting stretchable LED displays at 60 Hz refresh rates.

Bionic skin primarily achieves simulated tactile perception by integrating multimodal sensors to detect signals, including pressure and temperature. For instance, Wang et al. [131] developed a monolithically integrated low‐voltage soft electronic skin (e‐skin) multimodal sensor that successfully restored tactile perception. This sensor achieved low operating voltage through a three‐layer high‐dielectric‐constant elastomer dielectric design, converted stimulus amplitude into frequency‐modulated neural‐like pulse trains via ring oscillators and edge detector circuits, and utilized solid‐state synaptic transistors to trigger downstream muscle movements, ultimately establishing a closed‐loop biomimetic sensorimotor pathway from perception to actuation in an in vivo rat model. Dobashi et al. [132] developed a self‐powered piezoionic hydrogel array sensor that detects mechanical pressure distributions and emulates biological tactile perception. This sensor's operation is based on a material‐driven decoupling principle, utilizing pressure gradient‐induced ion migration to accurately quantify pressure magnitude, location, and temporal evolution (e.g., touch, compression, swiping), with adjustable signal duration (milliseconds to hundreds of seconds) and amplitude, successfully achieving neuromuscular modulation in an in vivo rat model. As demonstrated in Figure 15d, Dai et al. [133] invented a self‐adhesive multimodal electronic skin sensor based on a laser‐induced adhesive layer and a bio‐inspired 3D micro‐cilia architecture for simultaneously detecting pressure, strain, and bidirectional joint movements (inward flexion/outward extension), where the distinct mechanical deformation modes of the 3D micro‐cilia structure provide an intrinsic structural mechanism for signal decoupling, enabling the distinction of mechanical stimuli and human‐machine interaction (HMI) control through distinguishable resistance signals.

Furthermore, multimodal sensors find applications in human‐machine interaction contexts such as virtual reality and affective computing. Yu et al. [134] invented a skin‐integrated wireless tactile interface multimodal system for delivering virtual haptic stimuli to enable tactile interaction in virtual reality (VR) and augmented reality (AR). This system utilizes a wirelessly powered flexible e‐skin patch (featuring 32 independently controlled millimeter‐scale vibration actuators) that employs Lorentz force‐driven magnets to generate mechanical vibrations, programmably delivering localized tactile patterns on human skin for applications including social media‐enabled remote tactile interactions (e.g., transmitting remote touch), prosthetic control feedback (simulating object shapes via haptics), and gaming somatosensory interactions (impact feedback corresponding to body parts). As shown in Figure 15e, Pan et al. [135] developed a multimodal sensor system integrating facial expressions, speech signals, text, and physiological signals for detecting human discrete basic emotions (e.g., anger, disgust, fear, happiness, sadness, surprise) and dimensional emotions (e.g., valence‐arousal, pleasure‐arousal‐dominance), thereby enhancing emotion recognition accuracy through advanced algorithmic fusion of multi‐source information in human‐machine interaction.

These multimodal sensing breakthroughs redefine human‐machine interaction by surpassing biological perception limits and enabling closed‐loop bionic systems that restore sensorimotor functions. Through cross‐modal data fusion, they transform HMI from basic command execution to contextual environmental symbiosis—powering immersive virtual experiences, adaptive affective computing, and seamless human‐environment collaboration.

### 5.4 Intelligent Robotics

Building upon the sensing capabilities discussed previously, multimodal sensing has emerged as a core enabler for advanced robotic manipulation, facilitated by integrated environmental perception and closed‐loop control architectures. This technological convergence empowers robotic systems to achieve unprecedented precision in force feedback and thermal regulation during complex object interactions, thereby fundamentally enhancing task execution capabilities.

**FIGURE 16:**
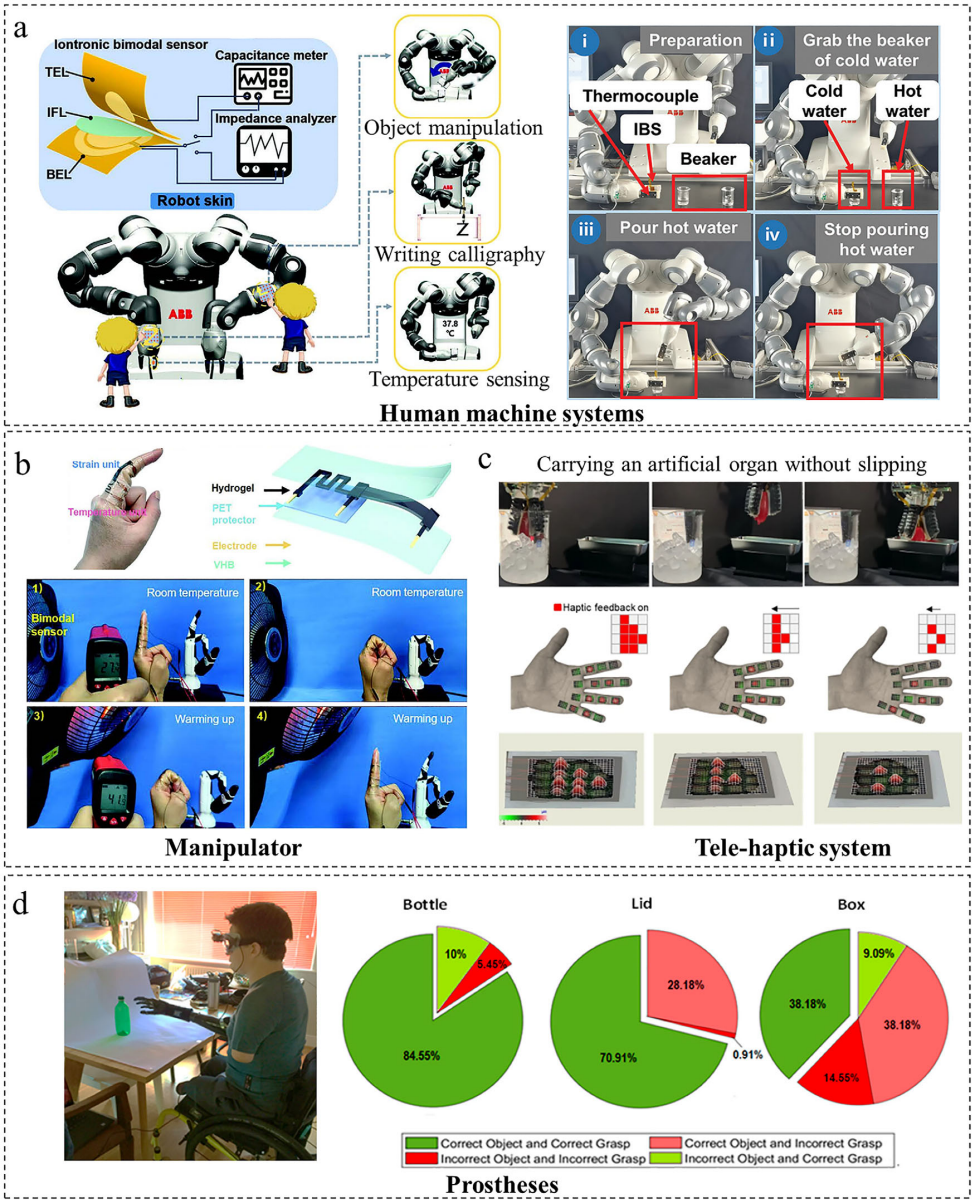
Applications of flexible multimodal sensors in intelligent robotics. (a) Application of multimodal sensors in human‐machine systems. Reproduced with permission [54]. Copyright 2023, WILEY‐VCH GmbH. (b) Bimodal sensor controlling the manipulator. Reproduced with permission [136]. Copyright 2023, WILEY‐VCH GmbH. (c) Tele‐haptic system demonstration. Reproduced with permission [137]. Copyright 2022, Springer Nature. (d) The amputee is carrying out the classification performance assessment on a bottle using a power grasp. Reproduced under terms of the CC‐BY Creative Commons Attribution 4.0 International License (http://creativecommons.org/licenses/by/4.0/) [138]. Copyright 2020, Gardner Marcus, published by MDPI.

In robotic applications, multimodal sensors enhance dexterous manipulation through refined force feedback and environmental cognition. Yang et al. [54] adopting a structural decoupling strategy though capacitive‐resistive dual‐mode measurements to separate pressure and temperature signals, featuring high sensitivity and a broad detection range (Figure 16a). When integrated into the inner side of an ABB YUMI robotic gripper, this sensor functioned as an “artificial thermal receptor” responding 30% faster than conventional thermocouples during the grasping of high‐temperature objects (e.g., 80°C metal components). Additionally, sensor arrays attached to the back of the robotic hand enabled real‐time parsing of hand movement directions (such as four‐way rotation), providing a tactile feedback foundation for remote surgical robots. Li et al. [136] invented a hydrogel‐based strain‐temperature bimodal sensor shown in Figure 16b for high‐precision detection of human motion strain signals and simultaneous elimination of temperature interference in dynamic temperature fields. When attached to robotic finger joints, the sensor monitors joint bending angles and ambient temperature changes in real time, feeding data back to the control system to enable stable execution of tasks such as grasping and assembly by robotic arms in complex thermal environments.

In addition, as illustrated in Figure 16c, Kwon et al. [137] developed a mechanically decoupled multimodal tactile sensor array (MDPS) that enables high‐precision detection of true contact pressure on dynamically curved soft surfaces such as human skin or soft robots through a dedicated mechanical design that isolates and eliminates deformation interference signals; in complex application scenarios, this technology achieves integrated monitoring of organ‐gripping pressure with accurate perception of directional characteristics in slippage events (recognition accuracy >99%) when deployed on soft robotic grippers. Gardner et al. [138] developed a multimodal sensing suite integrating mechanomyography (MMG), computer vision, and inertial measurement units (IMUs) to detect grasp intent in upper‐limb amputees for shared autonomy control of prostheses. This system's performance hinges on an algorithmic fusion strategy to decouple and classify intent from multi‐source sensory data, successfully achieving adaptive grasp classification for daily objects, with average classification accuracies of 96.17% (bottles), 76.97% (lids), and 86.41% (boxes), while significantly reducing calibration time and cognitive load shown in Figure 16d.

These innovations validate multimodal sensing as the essential infrastructure for next‐generation robotic manipulation, where integrated perception‐control architectures enable unprecedented operational precision. Through the synergistic fusion of force, thermal, and motion data, they elevate robotic capabilities from preprogrammed actions to contextually adaptive interactions in unstructured environments.

## 6 Conclusion and Outlook

To systematically compare and summarize the diverse decoupling strategies discussed, the key characteristics of material‐based, structural, and AI‐driven approaches are consolidated in Table 1. This table contrasts their core mechanisms, representative implementations, effectiveness in reducing crosstalk, and typical application scenarios. It provides a concise framework for understanding the trade‐offs and selection criteria among different decoupling pathways, highlighting that the choice of strategy fundamentally depends on the nature of the target stimuli, the required fidelity, and the constraints of the application environment.

**TABLE 1:** Summary and comparison table of decoupling strategies for multimodal sensors.

Decoupling strategy	Representative examples	Target stimuli	Decoupling mechanism	Remaining crosstalk	Advantages	Limitations	Application scenarios
Material design decoupling	[60]	pH, Na^+^, K^+^, Ca^2^^+^, temperature	Inherent material properties	Very low (drift <0.2 mV/h)	Intrinsic decoupling, algorithm‐free	Complex material design, limited scalability	Wearable sweat monitoring, real‐time health assessment
	[64]	Temperature, pressure	Composite materials design	Extremely low (near‐zero TEC)	Actively cancels interference, flexible design, robust	Complex multi‐component optimization	Smart textiles, health monitoring
	[67]	Pressure, material Type, airflow	Engineering of property differentials	Low (material classification accuracy 94%)	Single‐element multifunctionality, effective for complex fields	Relies on precise structure and signal processing	Robotic tactile perception, object identification
Structural design decoupling	[46]	Pressure, shear, bending, temperature	Microstructured architectures	Low (shear displacement resolution 500 µm, pressure sensitivity ‐0.1% kPa^−^^1^)	Hardware‐level force and temp. decoupling, high linearity	Specialized for force, complex fabrication	Dexterous robotics
	[79]	Temperature, strain	Planar designs	Very Low (Temperature error <±0.1 K under ±0.4% strain)	Hardware‐level isolation, high linearity and reliability.	complex structural design and fabrication	E‐skin, prosthetic haptics, structural health monitoring
	[83]	Temperature, humidity, pressure	3D stacking and integration	Very low (cross‐sensitivity <0.6%)	Simultaneous multi‐modal, minimal crosstalk	Complex fabrication, requires precise alignment	Wireless health monitoring
AI‐based Signal Decoupling	[48]	Tactile (X/Y/Z coordinates)	Shallow models	Model error (Z‐axis ∼1.41%)	Low‐cost, easy deployment	Limited capacity for complex coupling	Embedded tactile sensing, portable devices
	[49]	Temperature, pressure	Deep neural networks	Model error (pressure MAPE 1.58%)	Powerful nonlinear fitting, high accuracy	Demands large data and computation, costly	Intelligent grasping
	[112]	Strain, material, temperature	Hardware‐Software Co‐Design	Extremely low (<1% error)	Handles complex nonlinear coupling with high accuracy and adaptability.	Requires large labeled datasets; high computational cost	Intelligent robotic grasping, advanced wearables

With the growing demands on environment monitoring, health‐monitoring, human‐machine interaction, and intelligent robotics, have sparked rapid advancement in research on flexible multimodal sensors. Despite significant breakthroughs in physical mechanisms and materials design, structural design, and artificial intelligence strategies, achieving truly crosstalk‐free multidimensional sensing remains a major challenge, necessitating further fundamental research to fully unlock the potential of multimodal tactile sensing systems.

Effective decoupling, whose ultimate goal is the precise and reliable separation of target stimuli from entangled physical signals, serves as the foundational step toward reliable data fusion and advanced intelligence in next‐generation sensing systems. Future decoupling research for multimodal sensors involves three key directions:

Material‐based decoupling offers the intrinsic advantage of eliminating signal interference at the physical source through intrinsic physical phenomena and engineered material properties. However, it faces critical challenges where the inherent properties of materials limit their sensitivity and reliability. Future breakthroughs demand novel self‐decoupling materials, such as ionic materials [139], leveraging frequency‐dependent relaxation dynamics, offers a fundamental path to eliminating signal interference at its source.

Structural decoupling provides hardware‐level signal separation by mechanically isolating cross‐talk pathways through integrated physical segregation, strategic element placement, and localized stiffening. Its primary limitation lies in integration complexity with flexible substrates, so advancing structural design demands architectures like island‐bridge, serpentine, or anisotropic structures combined with inkjet or 3D printing techniques, to suppress cross‐talk and enhance detection capabilities for multiple stimuli interference.

AI‐driven decoupling delivers unparalleled precision in resolving machine learning algorithms for multimodal data decoupling and fusion, and artificial intelligence offers key advantages in overcoming current limitations by efficiently processing large, complex multimodal datasets. But physical crosstalk and system complexity remain fundamental challenges, so future advancements require algorithm optimization with deep integration of decoupled sensing systems and intelligent computing modules to eliminate multi‐parameter signal interference, thereby establishing robust foundations for practical AI‐decoupling technologies.

Looking ahead, the evolution of multimodal sensing systems hinges on fundamental advances in decoupling technologies that integrate novel materials, intelligent structures, and embedded intelligence. Future efforts should focus on developing self‐decoupling materials, innovative structural designs, and efficient embedded algorithms to achieve high‐fidelity, crosstalk‐free signal acquisition under realistic conditions. Such developments will critically support applications in high‐precision environmental monitoring [140], drive innovations in biomedical applications [141], and enable breakthroughs in human‐robot collaboration [142]. Ultimately, translating laboratory prototypes into real‐world solutions will require not only enhanced signal decoupling but also scalable manufacturing and seamless integration with edge computing, thereby enabling a new generation of intelligent systems capable of adaptive and reliable interaction with dynamic environments.

## Author Contributions

Y.L. W., Y.C., and Y.T.L. presented conceptualization, funding acquisition, review, and supervision. T.W. contributed to the investigation, original‐draft writing, and visualization. L.Y.Z., Y.Z., Z.J.Z., J.Y., Y.W.W., A.Q.C., Y.C., and Y.X.M. participated in checking the manuscript format. All authors discussed the results and commented on the manuscript.

## Conflicts of Interest

The authors declare no conflicts of interest.
